# Supplementary material for: Enantiocomplementary Bioreduction of 1-(Arylsulfanyl)propan-2-ones
Source: Molecules. 2024 Aug 15;29(16):3858. doi: 10.3390/molecules29163858 (PMC11357645; doi:10.3390/molecules29163858)
Supplement: Supplementary file 1 [file molecules-29-03858-s001.zip › molecules-3093981-supplementary.pdf]

molecules

MDPI

Enantiocomplementary bioreduction of 1-(arylsulfanyl)propan-2-ones

Emese Sándor [1], Pál Csuka [1], László Poppe [1*] and József Nagy [1*]

[1] Department of Organic Chemistry and Technology, Faculty of Chemical Technology and Biotechnology, Budapest University of Technology and Economics, H-1111 Budapest, Műegyetem rkp. 3, Hungary

* Correspondence: LP: poppe.laszlo.@vbk.bme.hu; NJ: nagy.jozsef@vbk.bme.hu

Table of contents

S1. Gas chromatography 2

S1.1. Temperature programs 2

S1.2. Molar response factors 2

S1.3. Gas chromatograms of the starting materials and products 3

S1.4. Gas chromatograms for checking the enantiomeric composition of the products 6

S2. Spectra of the starting materials and products 8

S1. Gas chromatography

S1.1. *Temperature programs*

Table S1. GC methods and retention times of **2a-e**, (±) **1a-e** and (*S*)- and (*R*)-**1d** and (*S*)- and (*R*)-**5a-c,e** to determine conversion and enantiomeric composition.

**Substrate**	**Method**	**Retention time (min)**			
		**2**	**(±)-1**	**(*R*)-5**	**(*S*)-5**
**2a**	160 °C, 5 min; 5 °C min^{-1} to 200 °C; 200 °C, 1 min	4.3	5.3		
**2a**	120 °C, 7 min; 0.8 °C min^{-1} to 140 °C			10.4	10.6
**2b**	160 °C, 5 min; 5 °C min^{-1} to 200 °C; 1 min	6.2	7.2		
**2b**	110 °C, 5 min; 0.8 °C min^{-1} to 130 °C			21.4	21.7
**2c**	170 °C, 3 min; 4 °C min^{-1} to 200 °C; 3 min	6.5	7.7		
**2c**	120 °C, 7 min; 0.8 °C min^{-1} to 140 °C			26.4	26.7
**2d**	170 °C 3 min; 4 °C min^{-1} to 200 °C; 3 min	8.9	10.5		
**2d**	150 °C, 20 min; 0.8 °C min^{-1} to 175 °C; 5 °C min^{-1} to 190 °C			36.9[1]	37.4[1]
**2e**	160 °C, 5 min; 5 °C min^{-1} to 200 °C; 1 min	9.9	10.9		
**2e**	120 °C, 7 min; 0.8 °C min^{-1} to 140 °C			28.3	28.6

[1]In the case of (*R*)-**1d** and (S)-**1d**, derivatization was not necessary.

S1.2. *Molar response factors*

To determine molar response factors for FID signals in GC, chromatograms of calibration samples from synthetically prepared (±)-**1a-e** and **2a-e** in 1:1 molar ratio were determined. The calculated molar response factors are shown in Table S2.

Table S2. Molar response factors of alcohols **1a-e** to ketones **2a-e**.

Ketone	Response factor
**2a**	0.9893
**2b**	1.0250
**2c**	1.0457
**2d**	0.7280
**2e**	0.9122

S1.3. Gas chromatograms of the starting materials and products

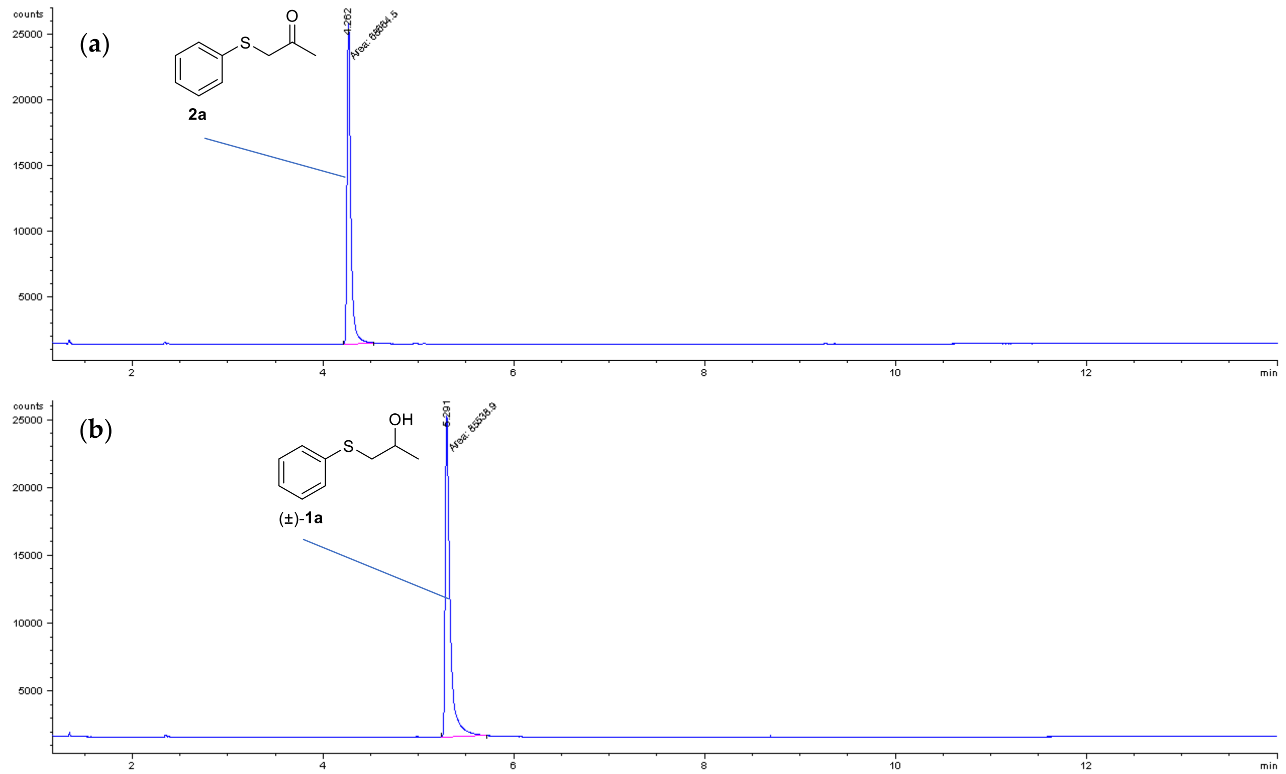

Figure S1. Chromatogram of (**a**) 1-(phenylsulfanyl)propan-2-one **2a** and (**b**) 1-(phenylsulfanyl)propan-2-ol (**±**)-**1a**.

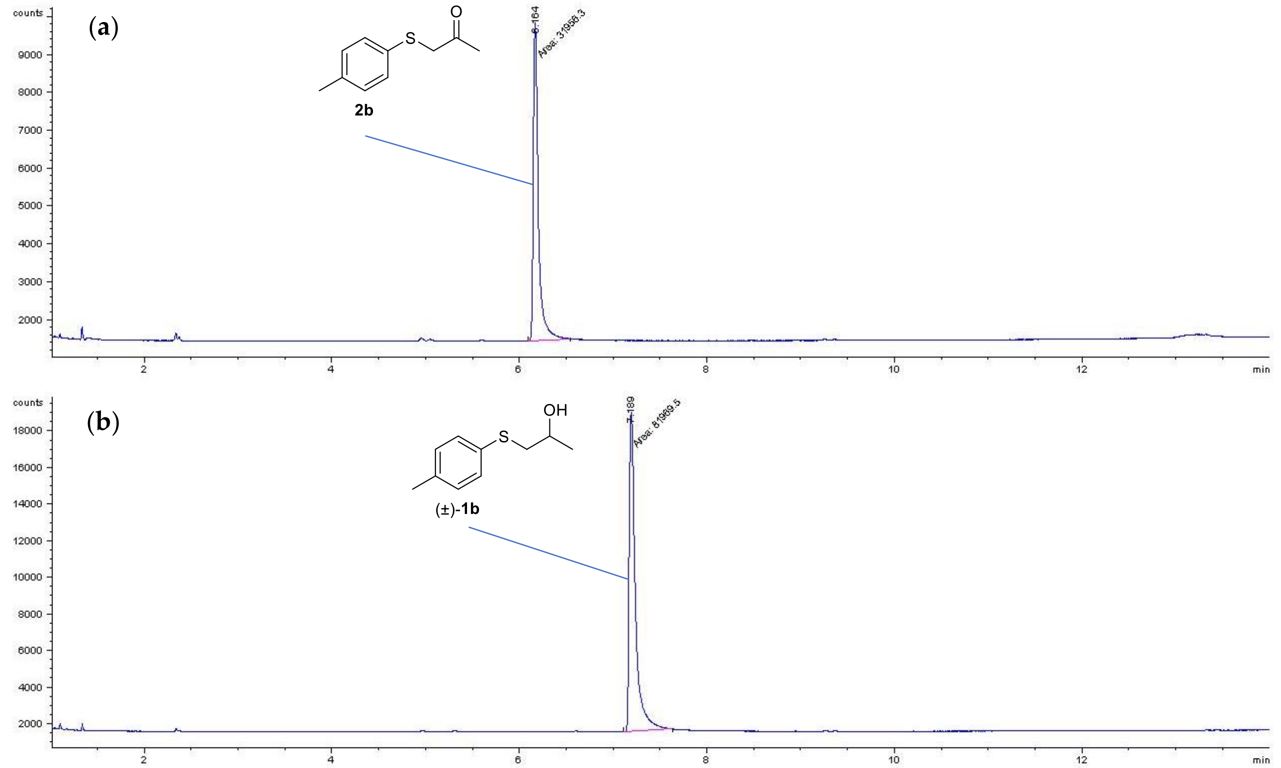

Figure S2. Chromatogram of (**a**) 1-[(4-methylphenyl)sulfanyl]propan-2-one (**2b**) and (**b**) 1-[(4-methylphenyl)sulfanyl]propan-2-ol (±)-**1b**.

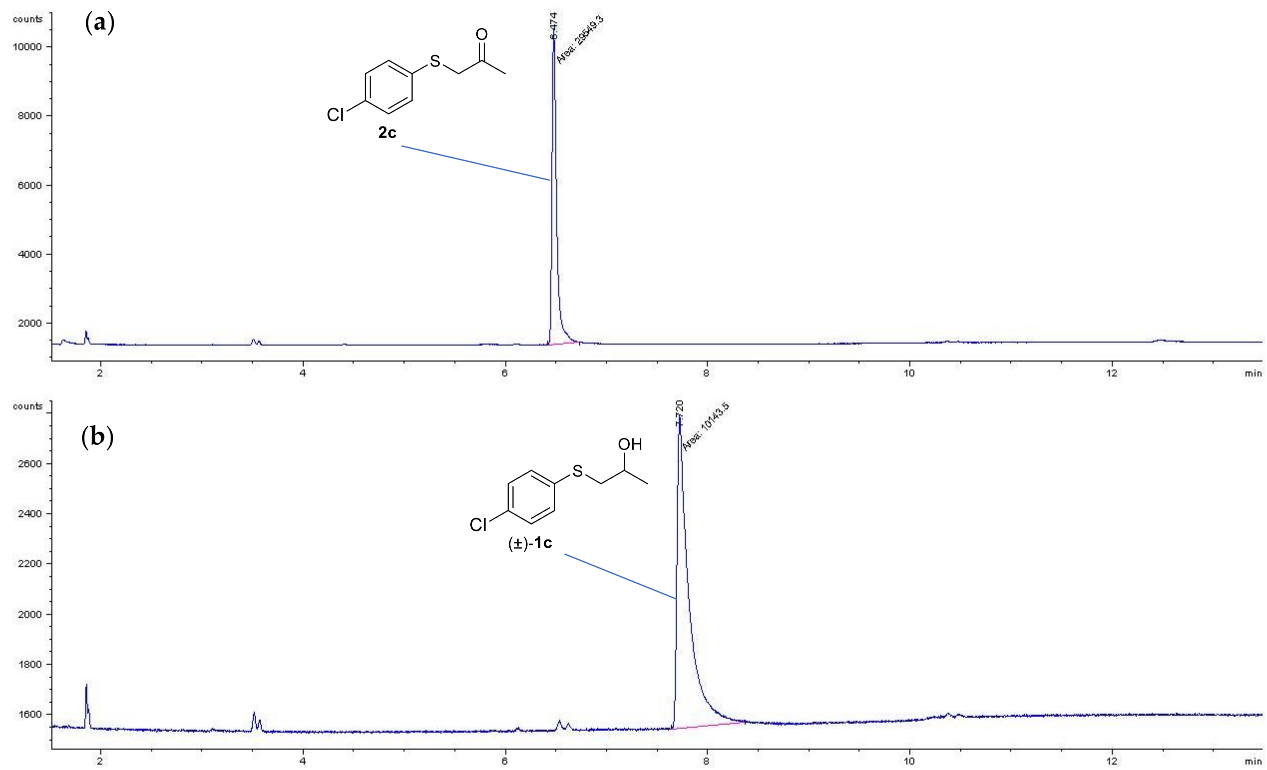

Figure S3. Chromatogram of (**a**) 1-[(4-chlorophenyl)sulfanyl]propan-2-one (**2c**) and (**b**) 1-[(4-chlorophenyl)sulfanyl]propan-2-ol (**±)-1c**.

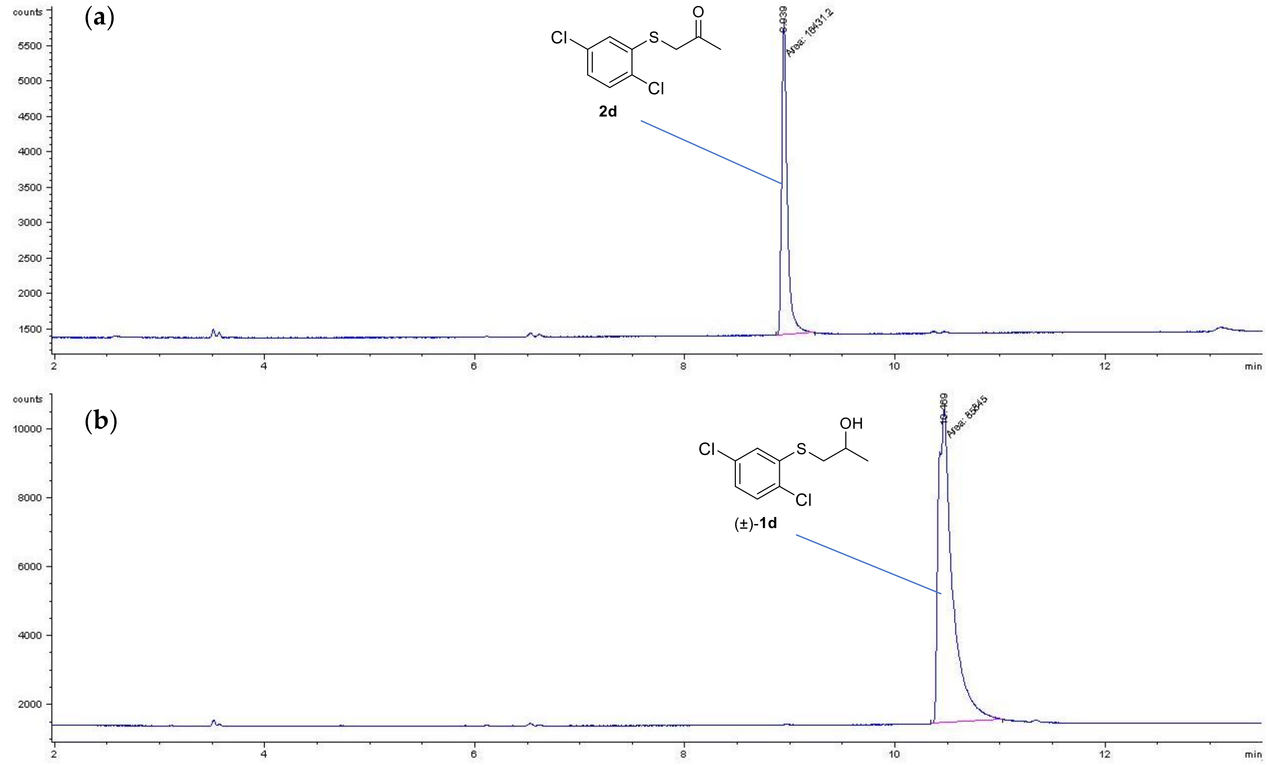

Figure S4. Chromatogram of (**a**) 1-[(2,5-dichlorophenyl)sulfanyl]propan-2-one **2d** and (**b**) 1-[(2,5-dichlorophenyl)sulfanyl]propan-2-ol (**±)-1d**.

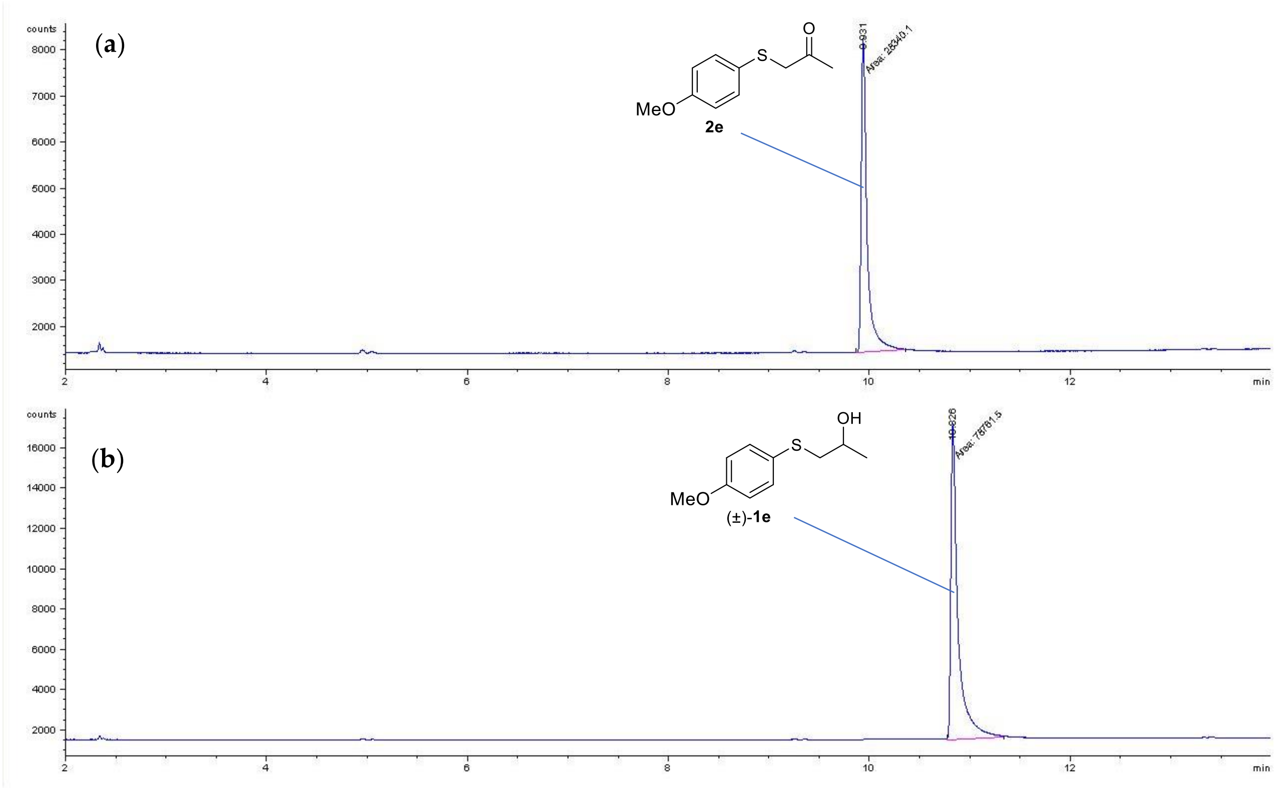

Figure S5. Chromatogram of (**a**) 1-[(4-methoxyphenyl)sulfanyl]propan-2-one (**2e**) and (**b**) 1-[(4-methoxyphenyl)sulfanyl]propan-2-ol (**±)-1e**.

S1.4. Gas chromatograms for checking the enantiomeric composition of the products

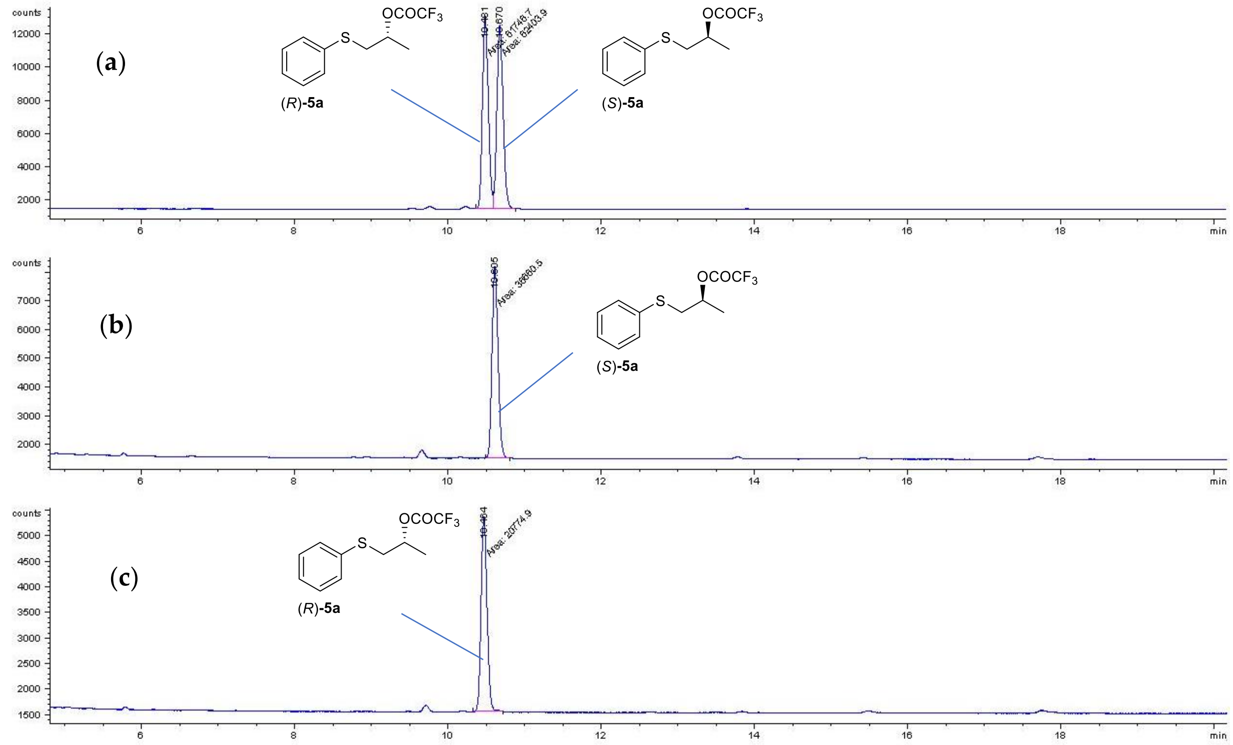

Figure S6. Chromatogram of (**a**) racemic (±)-1-(phenylsulfanyl)propan-2-yl trifluoroacetate [(*R*)-**5a** and (*S*)-**5a**]; (**b**) derivatized sample from bioreduction of 1-(phenylsulfanyl)propan-2-one **2a** with WY12 after 24 h and (**c**) from bioreduction with LkADH after 24 h.

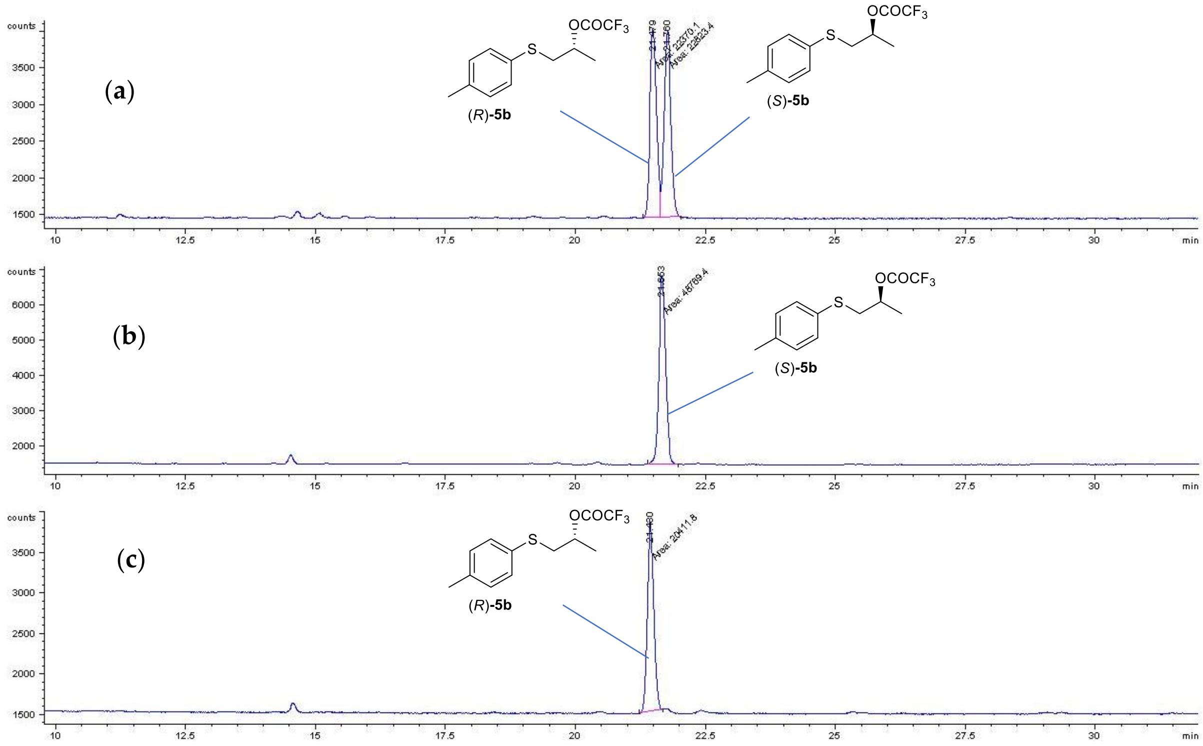

Figure S7. Chromatogram of (**a**) racemic (±)-1-[(4-methylphenyl)sulfanyl]propan-2-yl trifluoroacetate [(*R*)-**5b** and (*S*)-**5b**]; (**b**) derivatized sample from bioreduction of 1-[(4-methylphenyl)sulfanyl]propan-2-one **2b** with WY12 after 24 h and (**c**) from bioreduction with LkADH after 24 h.

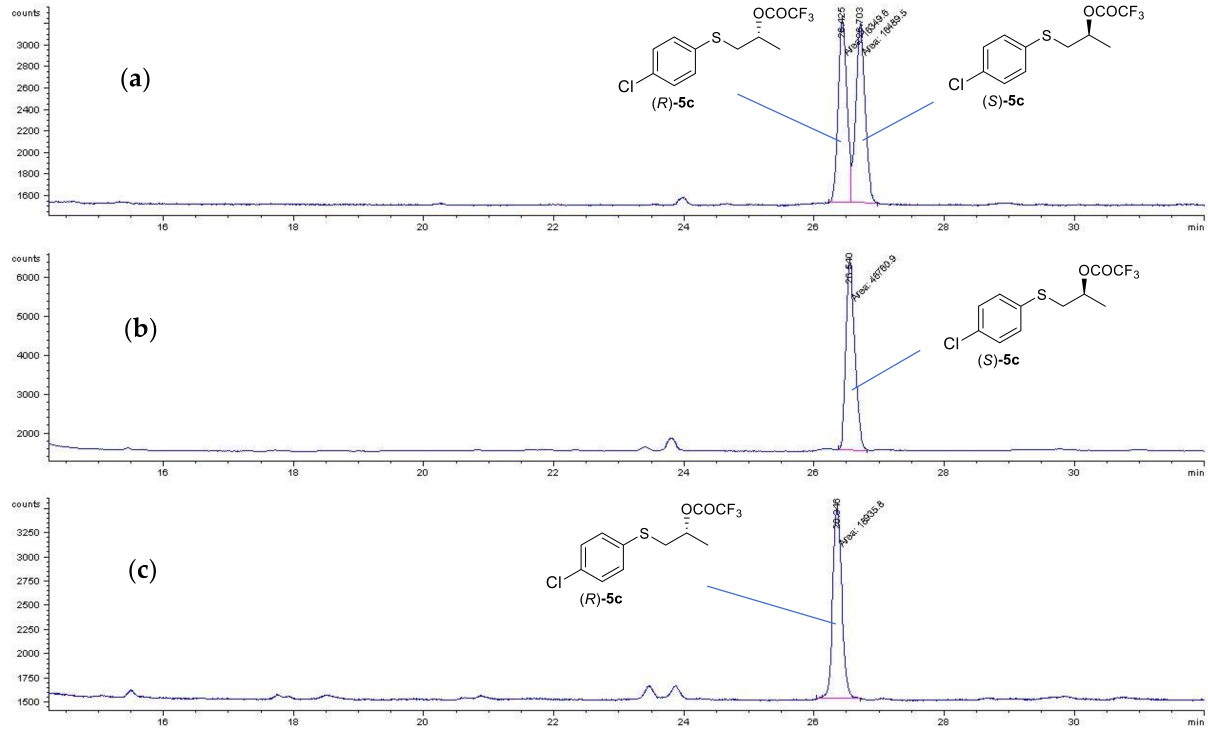

Figure S8. Chromatogram of (**a**) racemic (±)-1-[(4-chlorophenyl)sulfanyl]propan-2-yl trifluoroacetate [(*R*)-**5c** and (*S*)-**5c**]; (**b**) derivatized sample from bioreduction of 1-[(4-chlorophenyl)sulfanyl]propan-2-one **2c** with WY12 after 24 h and (**c**) from bioreduction with LkADH after 24 h.

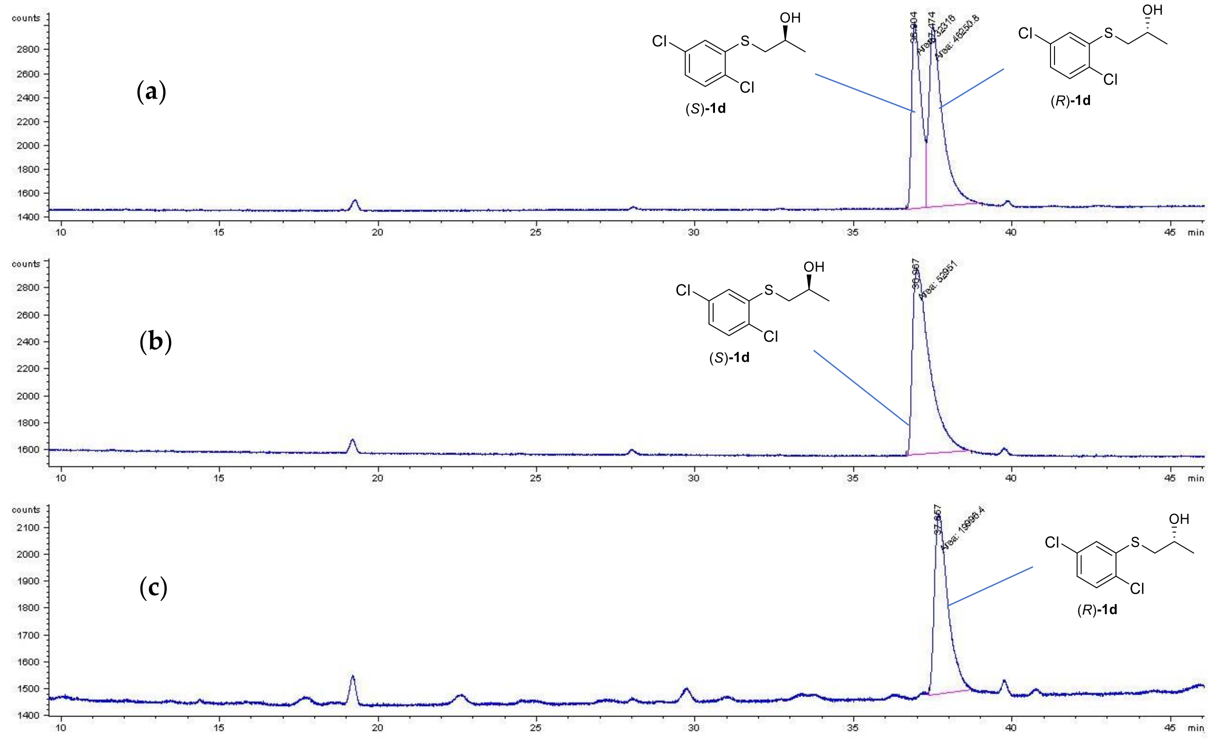

Figure S9. Chromatogram of (**a**) (racemic (±)-1-[(2,5-dichlorophenyl)sulfanyl]propan-2-ol [(*S*)-**1d** and (*R*)-**1d**]; (**b**) sample from bioreduction of 1-[(2,5-dichlorophenyl)sulfanyl]propan-2-one **2c** with WY12 after 24 h and (**c**) from bioreduction with LkADH after 24 h.

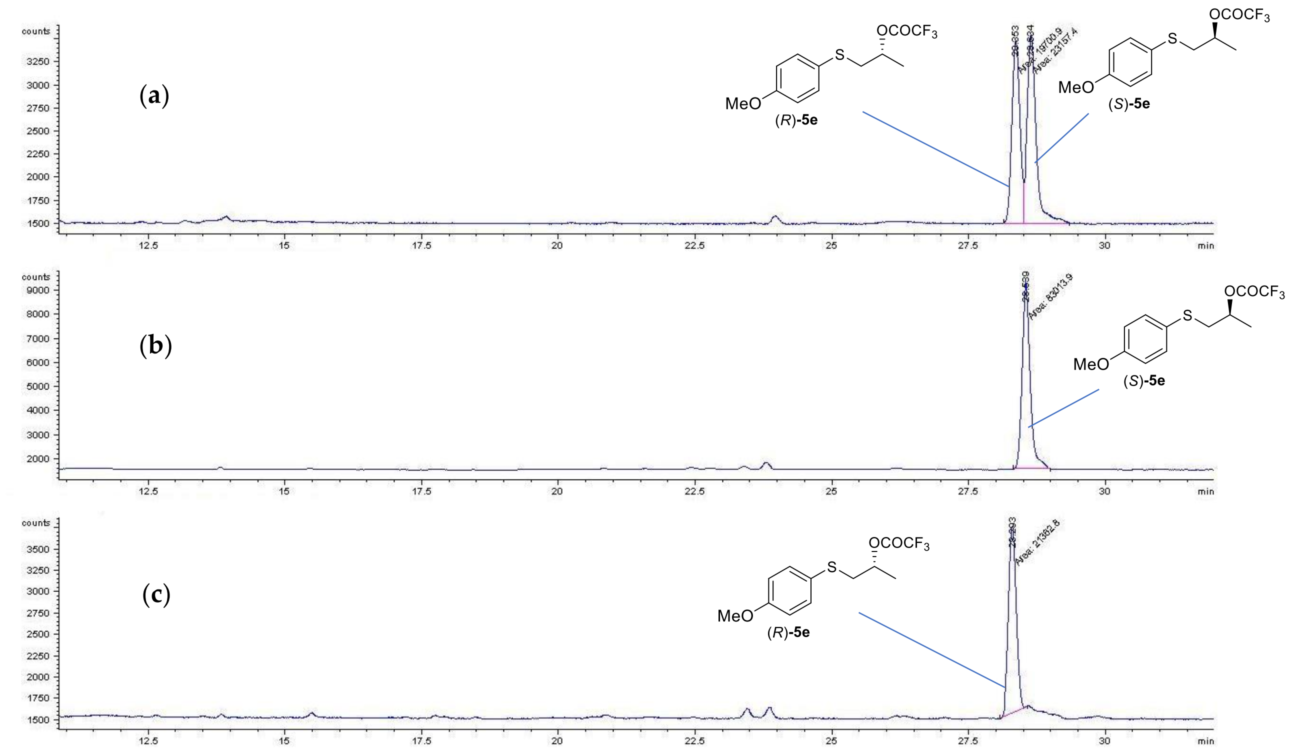

Figure S10. Chromatogram of (**a**) racemic (±)-1-[(4-methoxyphenyl)sulfanyl]propan-2-yl trifluoroacetate [(*R*)-**5e** and (*S*)-**5e**]; (**b**) derivatized sample from bioreduction of 1-[(4-methoxyphenyl)sulfanyl]propan-2-one **2e** with WY12 after 24 h and (**c**) from bioreduction with LkADH after 24 h.

S2. Spectra of the starting materials and products

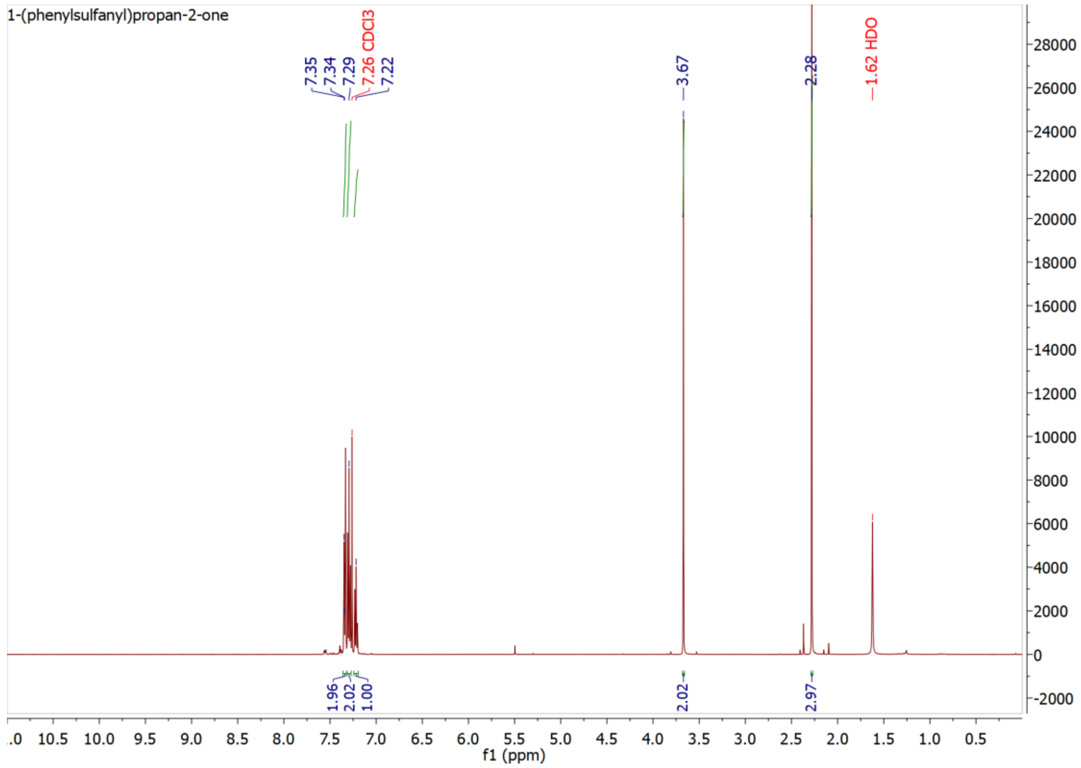

Figure S11. ^{1}H NMR spectrum of 1-(phenylsulfanyl)propan-2-one **2a**.

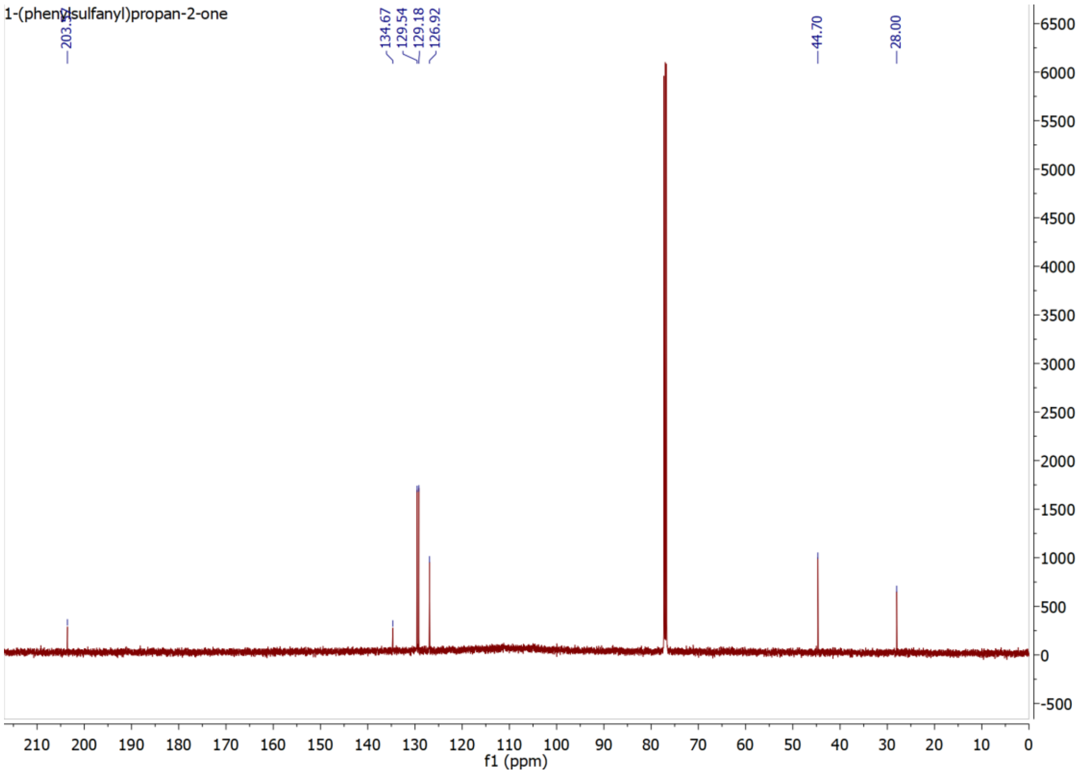

Figure S12. ^{13}C NMR spectrum of 1-(phenylsulfanyl)propan-2-one **2a**.

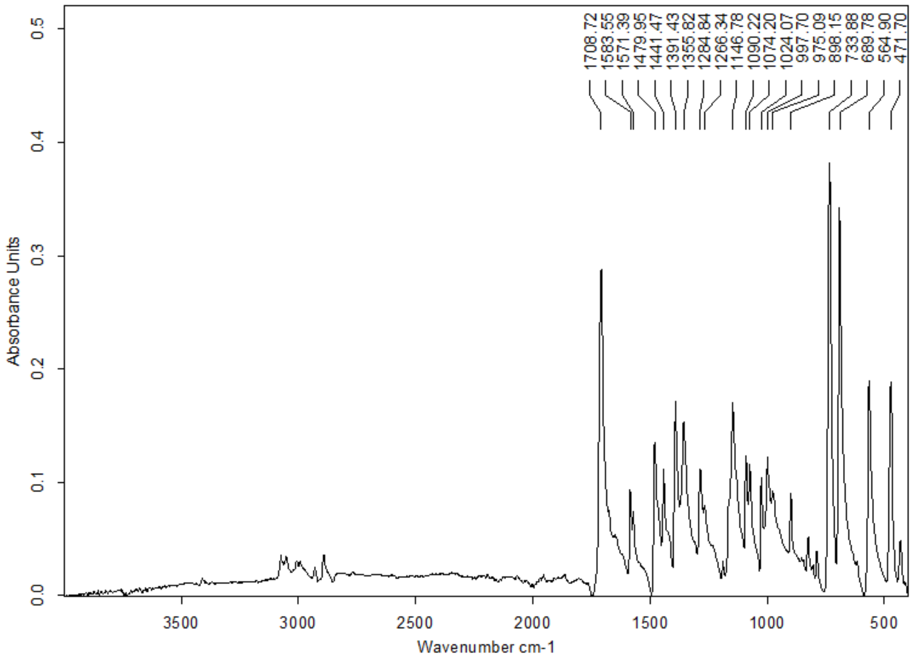

Figure S13. IR spectrum of 1-(phenylsulfanyl)propan-2-one **2a**.

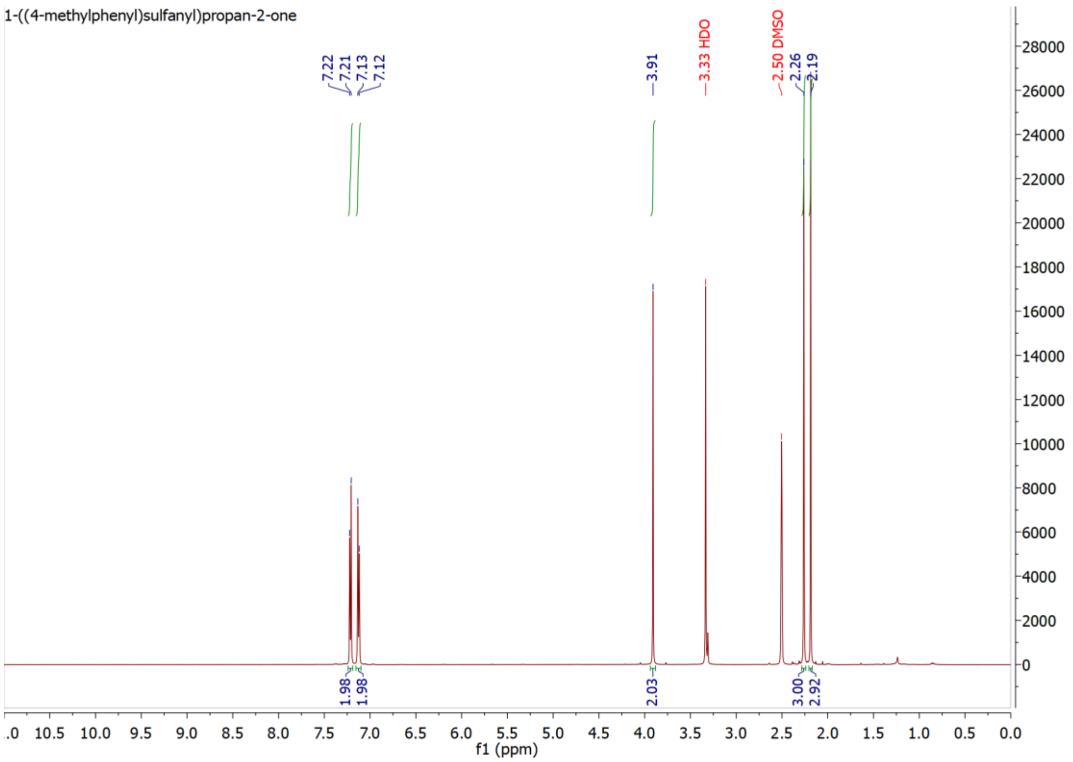

Figure S14. ^{1}H NMR spectrum of 1-[(4-methylphenyl)sulfanyl]propan-2-one **2b**.

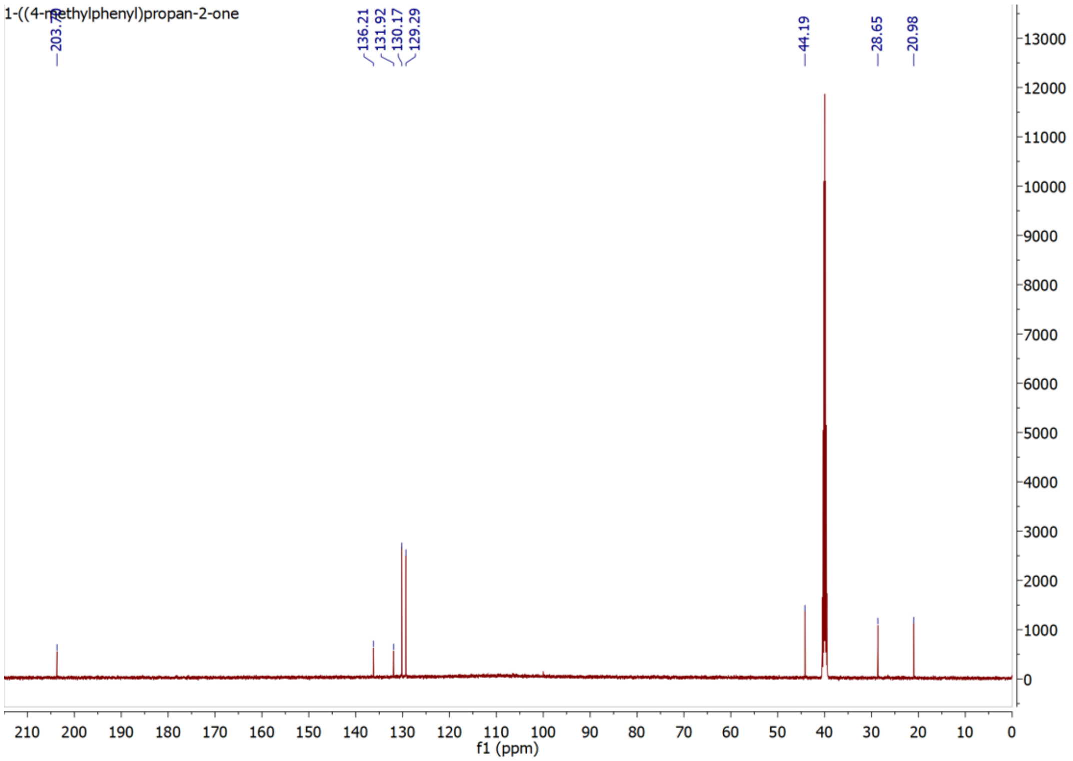

Figure S15. ^{13}C NMR spectrum of 1-[(4-methylphenyl)sulfanyl]propan-2-one **2b**.

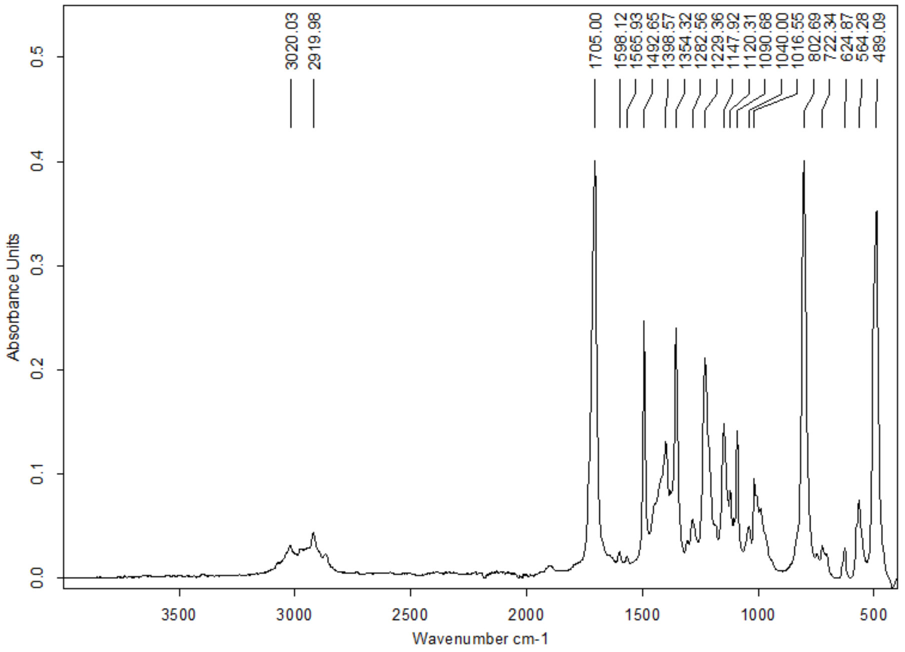

Figure S16. IR spectrum of 1-[(4-methylphenyl)sulfanyl]propan-2-one **2b**.

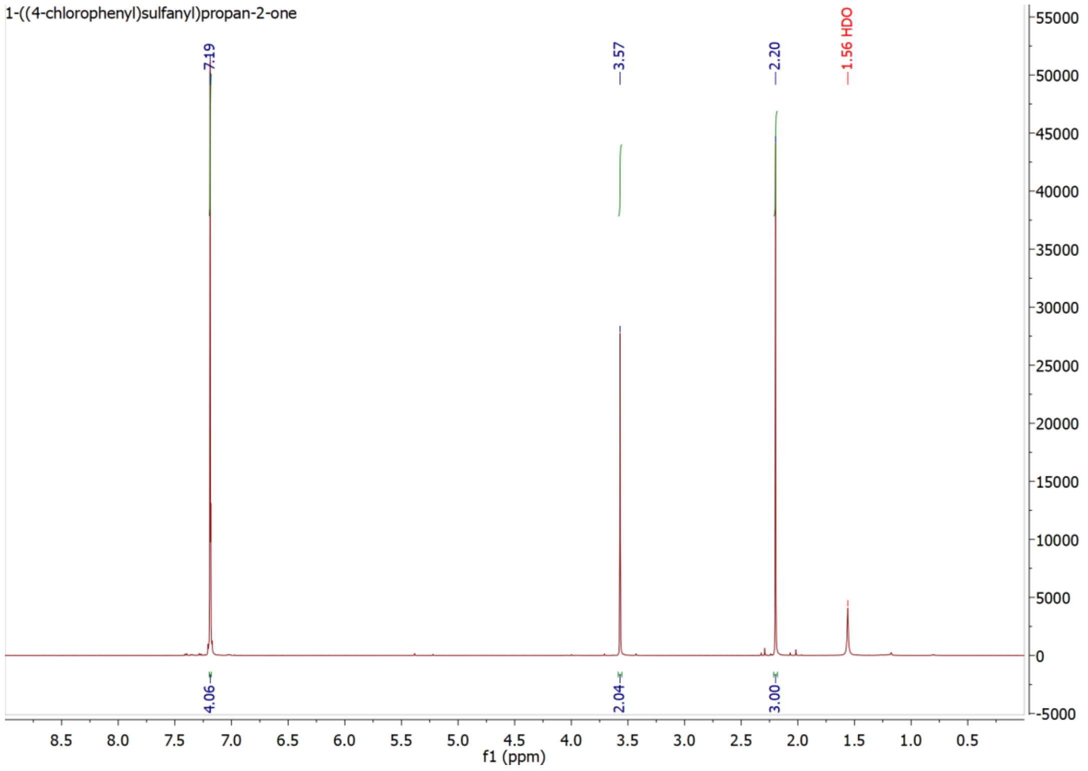

Figure S17. ¹H NMR spectrum of 1-[(4-chlorophenyl)sulfanyl]propan-2-one **2c**.

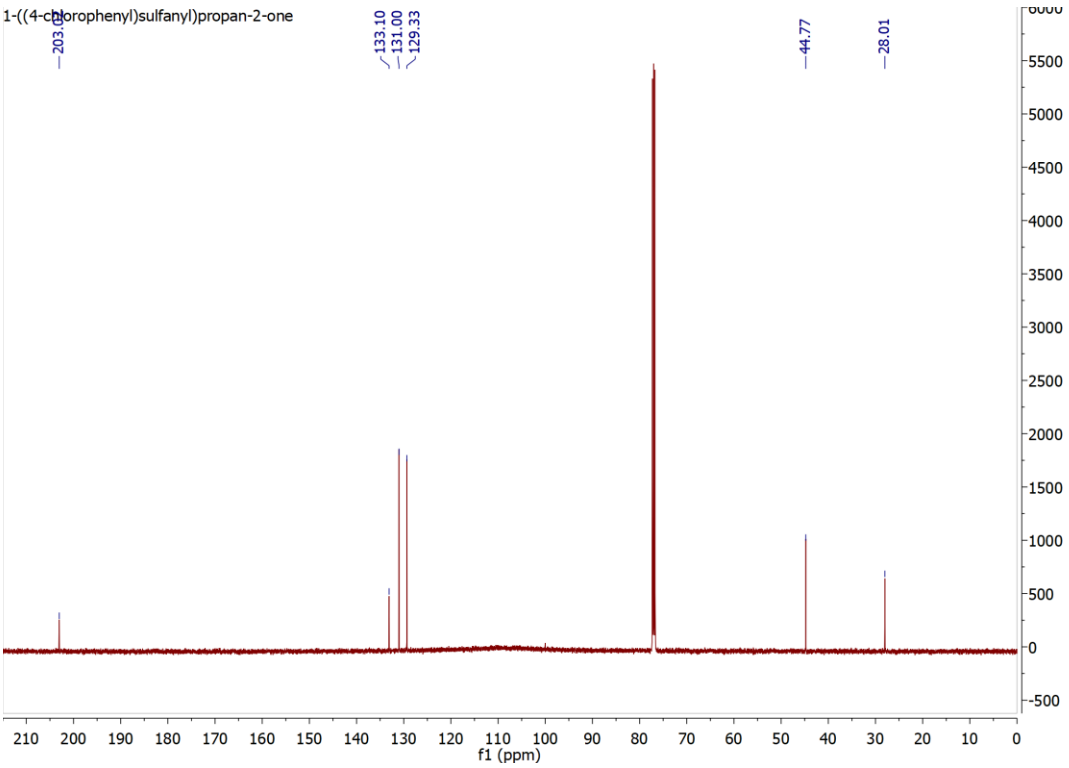

Figure S18. ^{13}C NMR spectrum of 1-[(4-chlorophenyl)sulfanyl]propan-2-one **2c**.

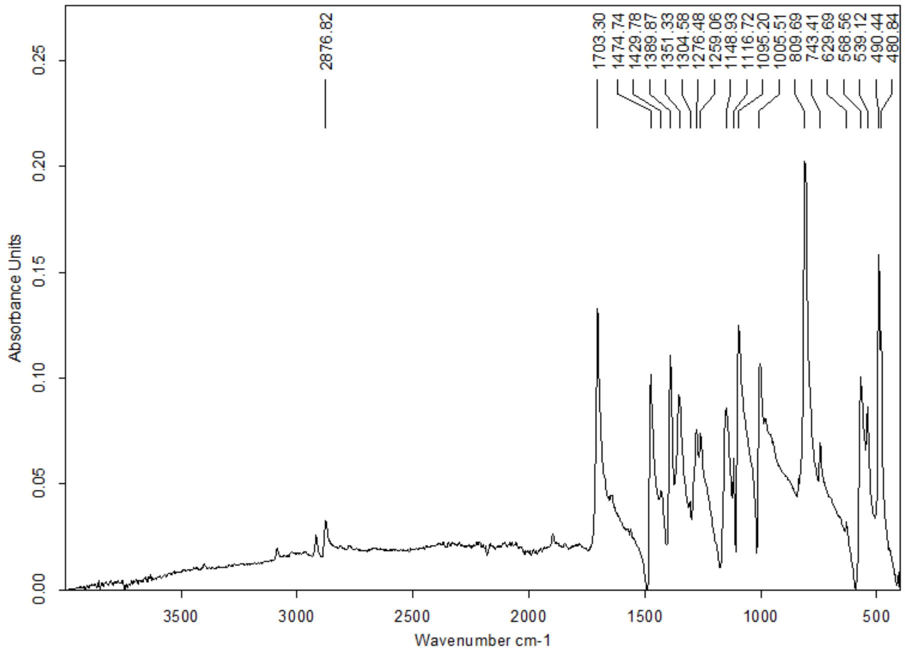

Figure S19. IR spectrum of 1-[(4-chlorophenyl)sulfanyl]propan-2-one **2c**.

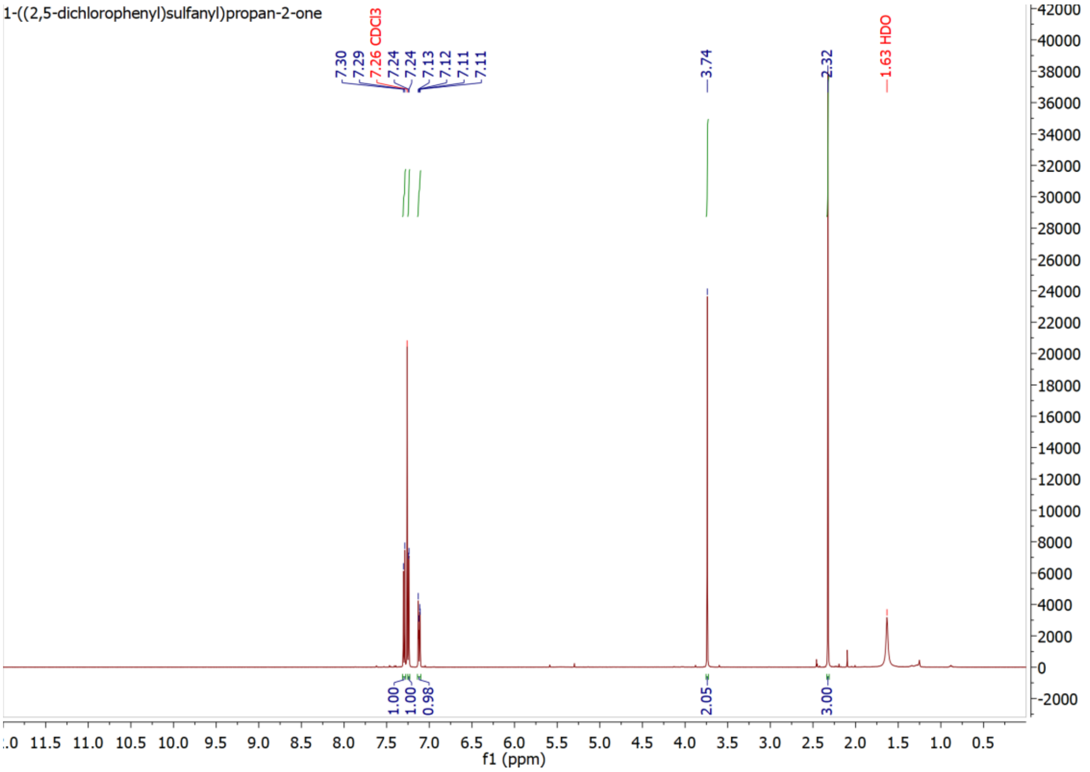

Figure S20. ^{1}H NMR spectrum of 1-[(2,5-dichlorophenyl)sulfanyl]propan-2-one **2d**.

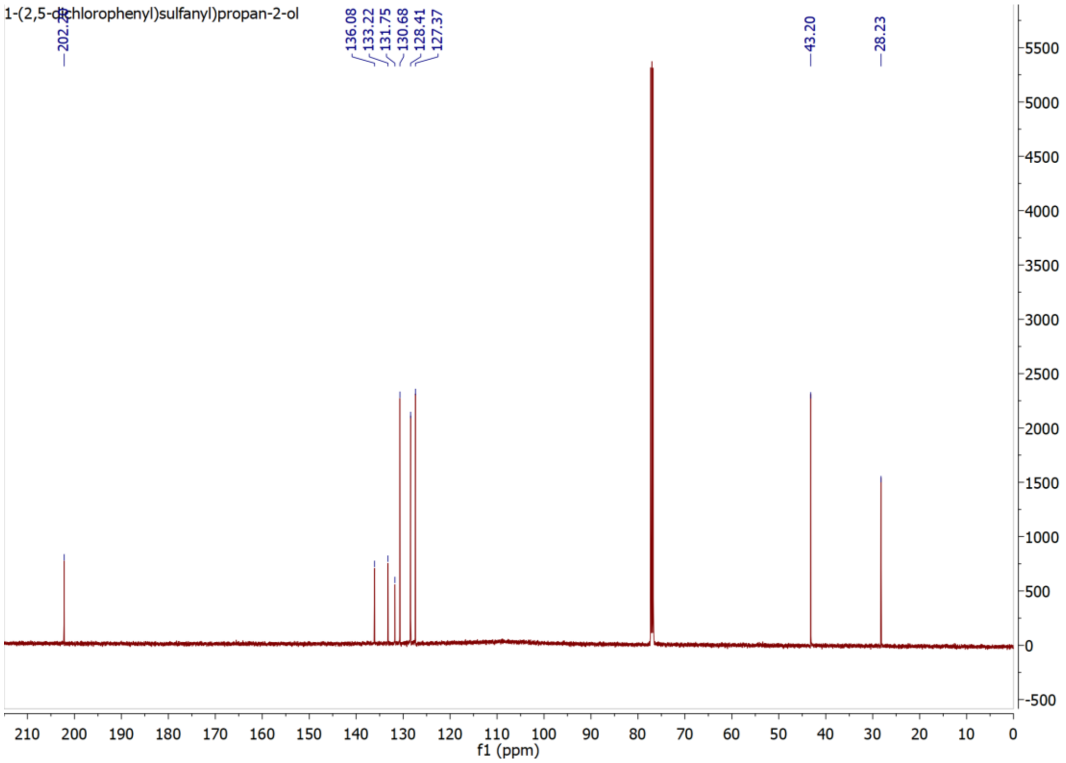

Figure S21. ^{13}C NMR spectrum of 1-[(2,5-dichlorophenyl)sulfanyl]propan-2-one **2d**.

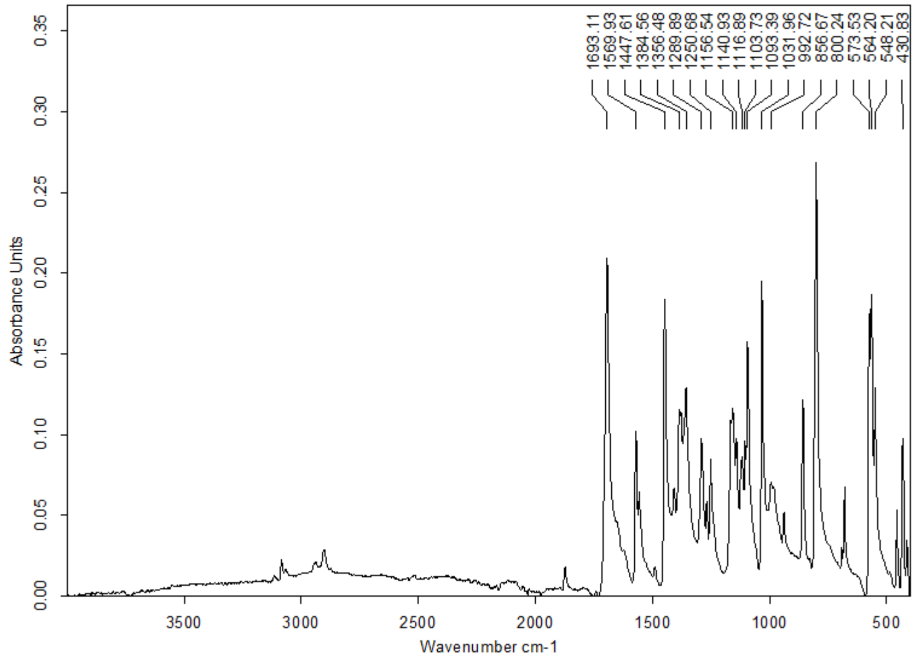

Figure S22. IR spectrum of 1-[(2,5-dichlorophenyl)sulfanyl]propan-2-one **2d**.

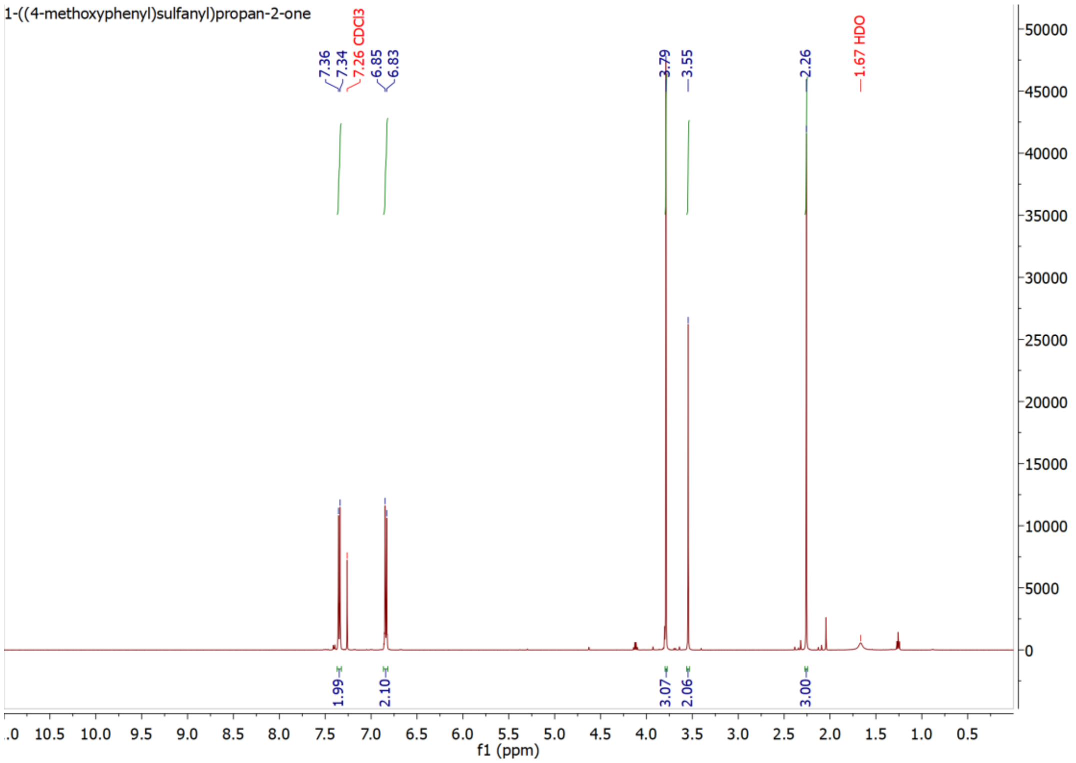

Figure S23. ^{1}H NMR spectrum of 1-[(4-methoxyphenyl)sulfanyl]propan-2-one **2e**.

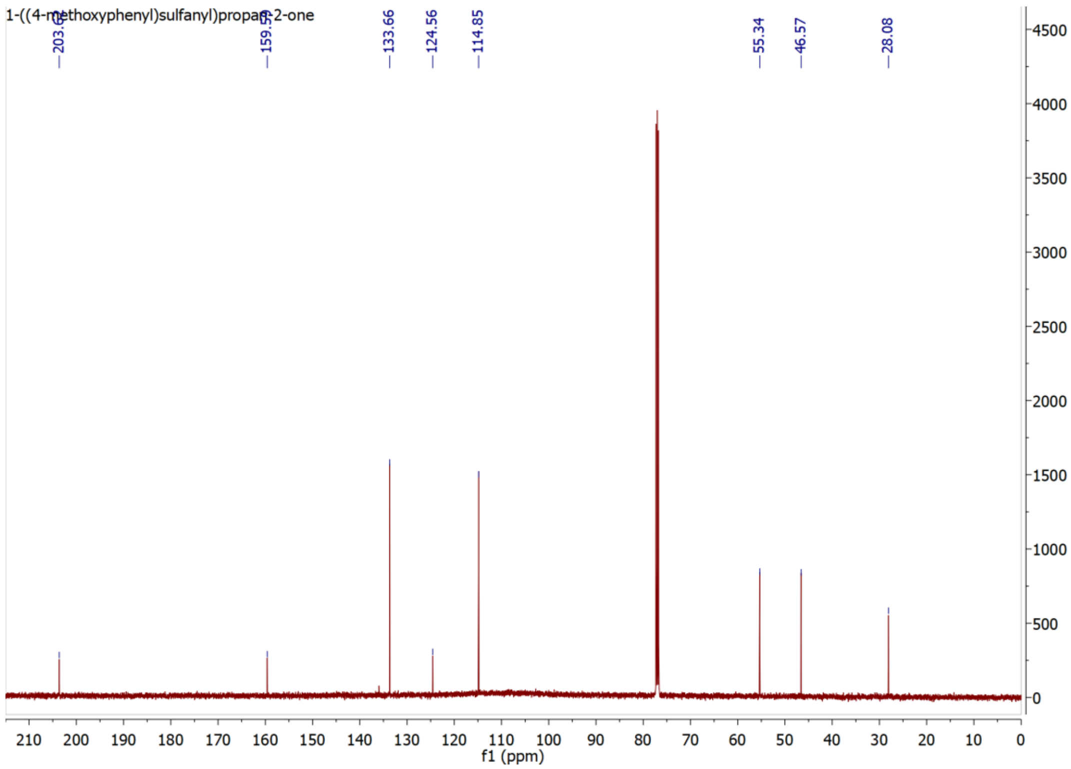

Figure S24. ^{13}C NMR spectrum of 1-[(4-methoxyphenyl)sulfanyl]propan-2-one **2e**.

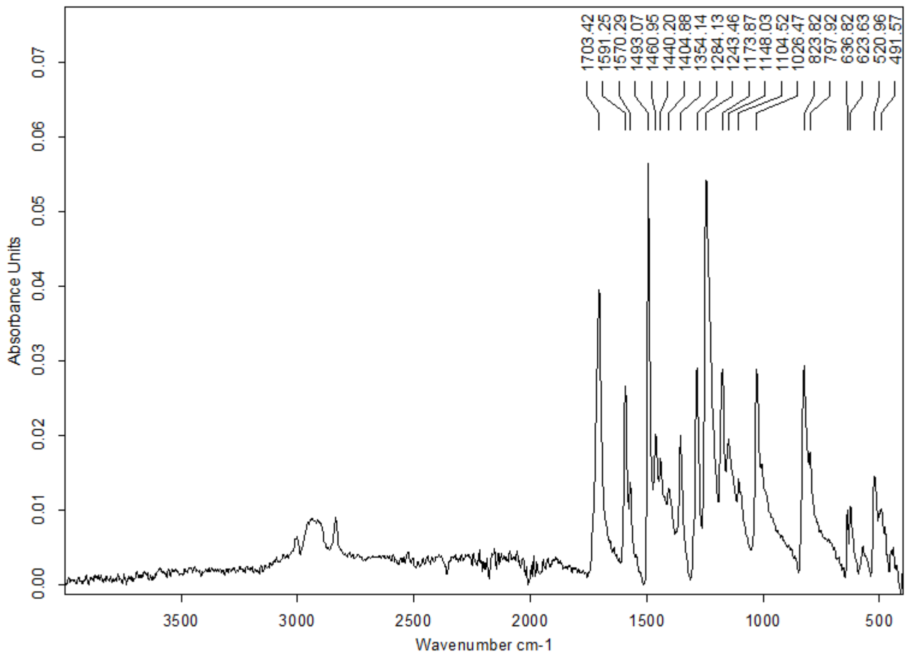

Figure S25. IR spectrum of 1-[(4-methoxyphenyl)sulfanyl]propan-2-one **2e.**

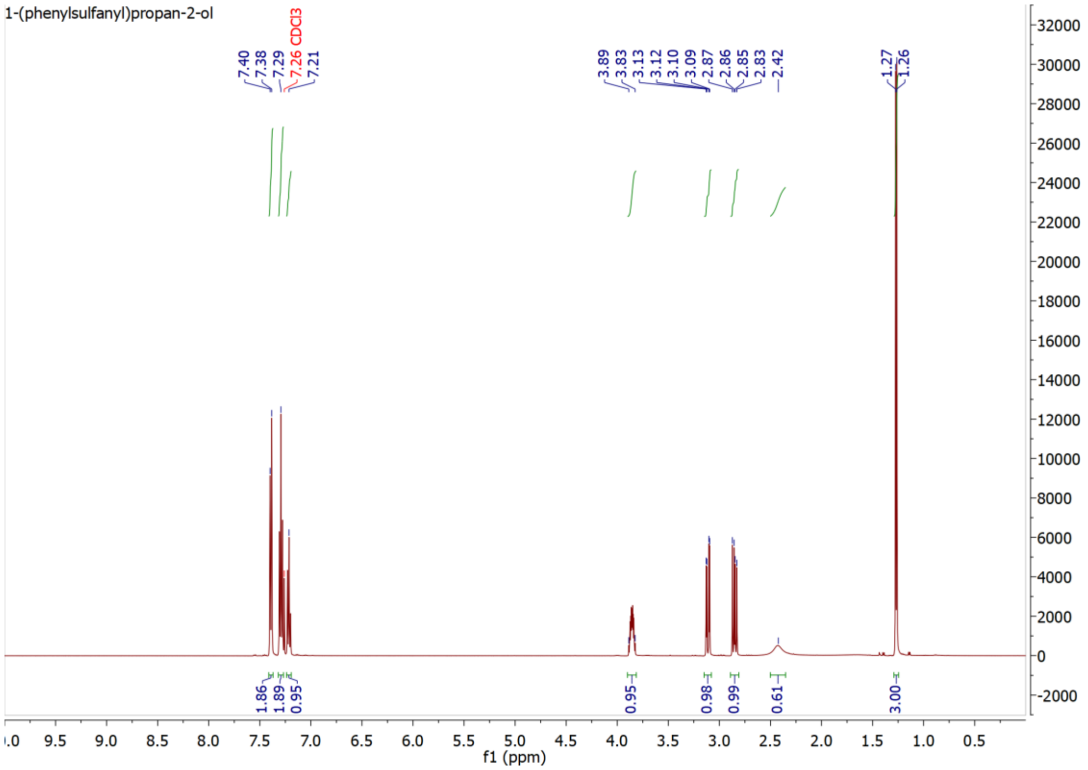

Figure S26. ^{1}H NMR spectrum of racemic 1-(phenylsulfanyl)propan-2-ol (±)-**1a**.

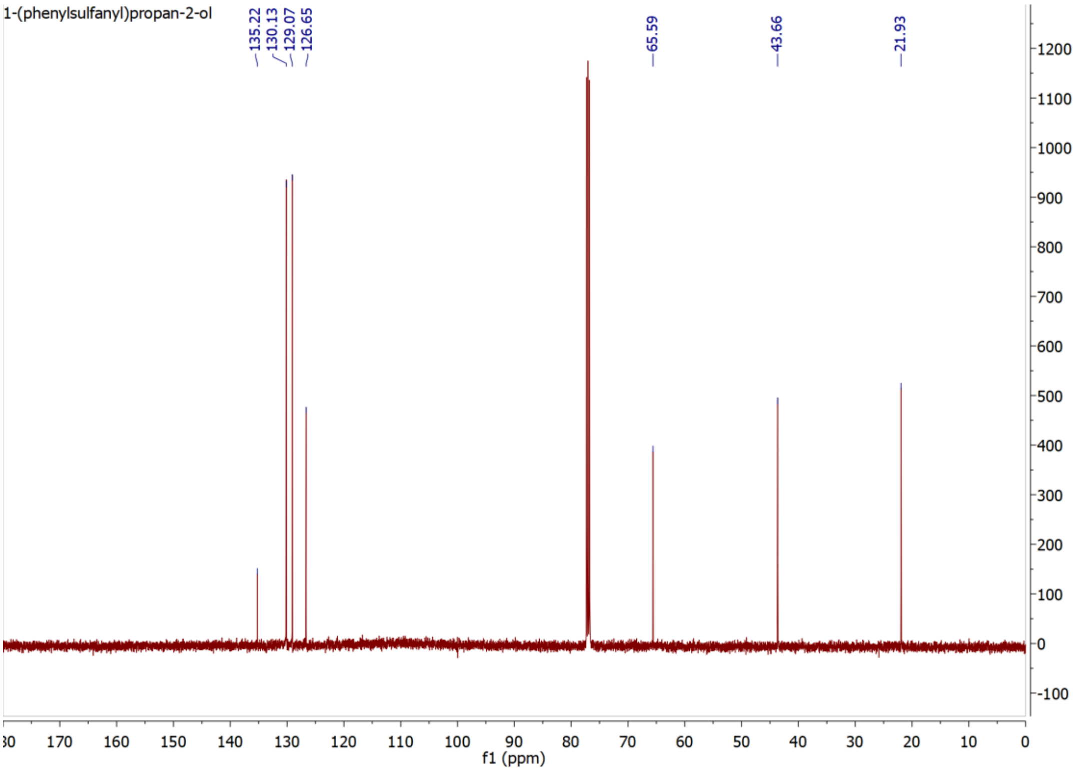

Figure S27. ^{13}C NMR spectrum of racemic 1-(phenylsulfanyl)propan-2-ol (±)-**1a**.

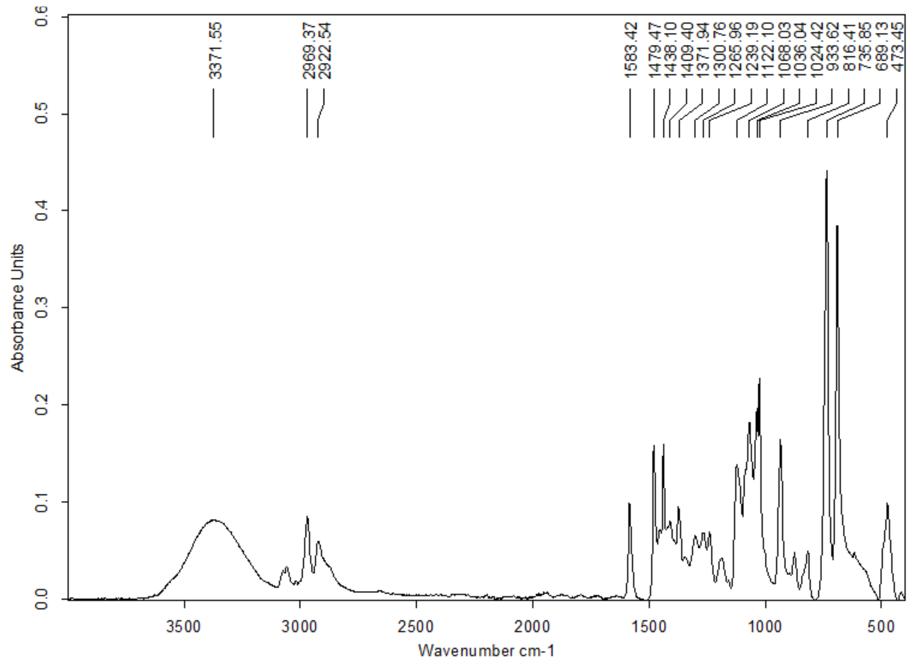

Figure S28. IR spectrum of racemic 1-(phenylsulfanyl)propan-2-ol (±)-**1a**.

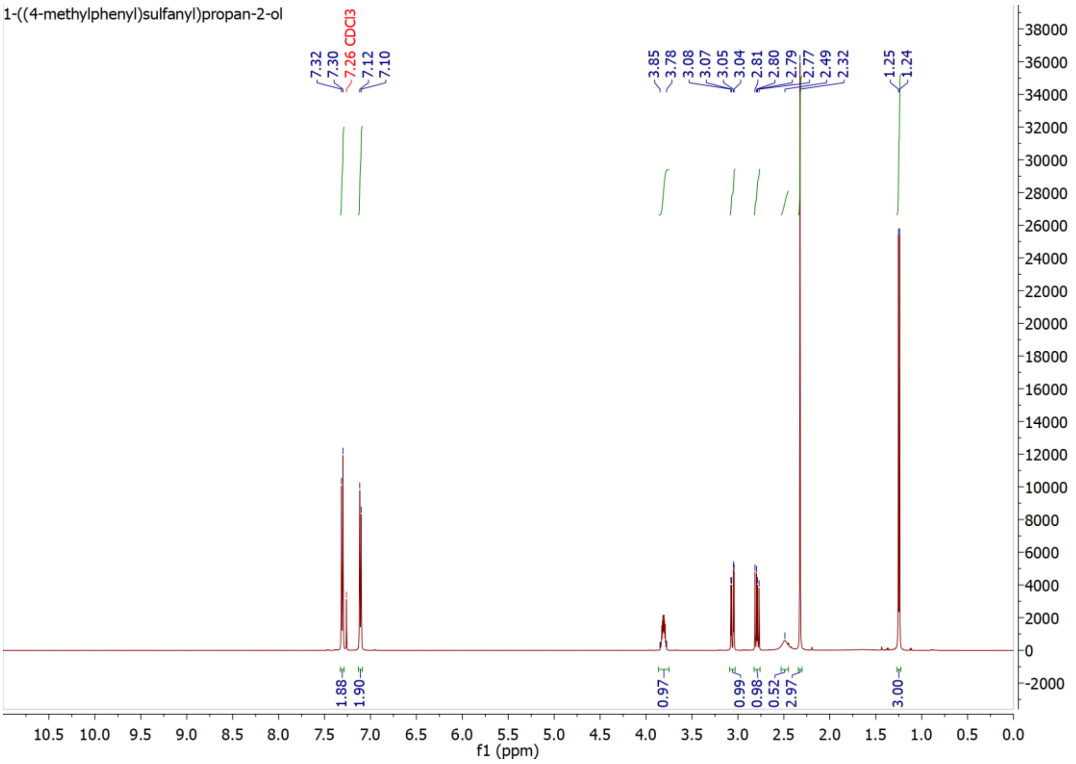

Figure S29. ^{1}H NMR spectrum of racemic 1-[(4-methylphenyl)sulfanyl]propan-2-ol (±)-**1b**.

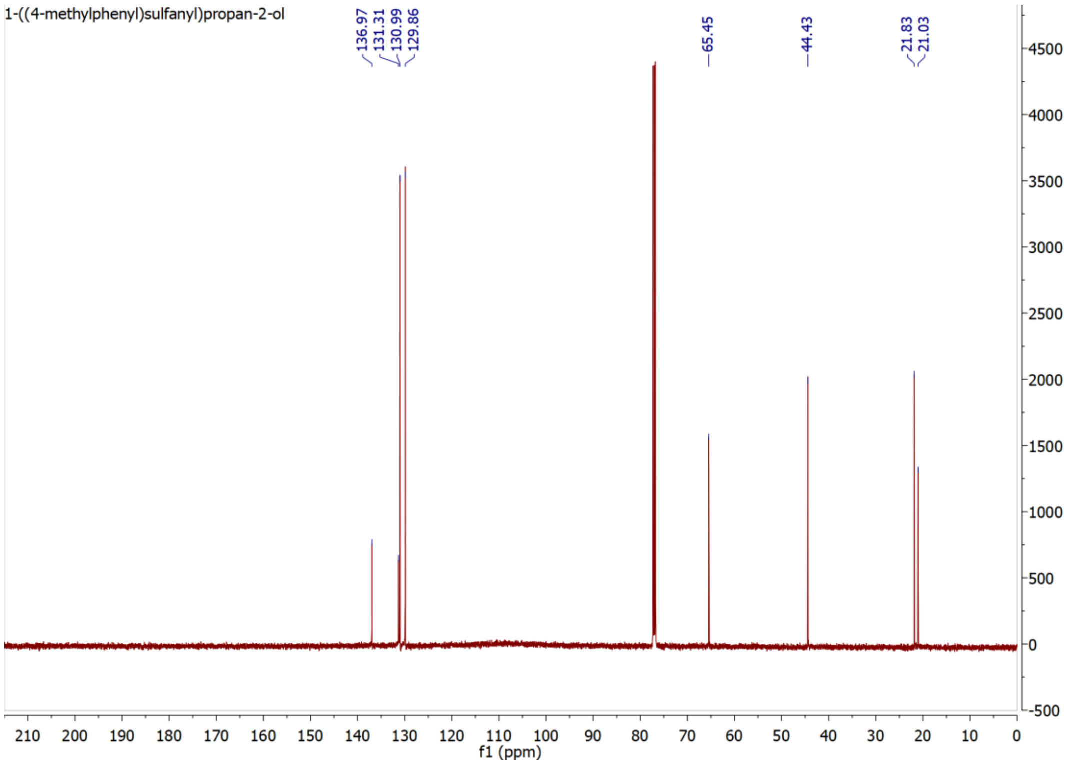

Figure S30. ^{13}C NMR spectrum of racemic 1-[(4-methylphenyl)sulfanyl]propan-2-ol (±)-**1b**.

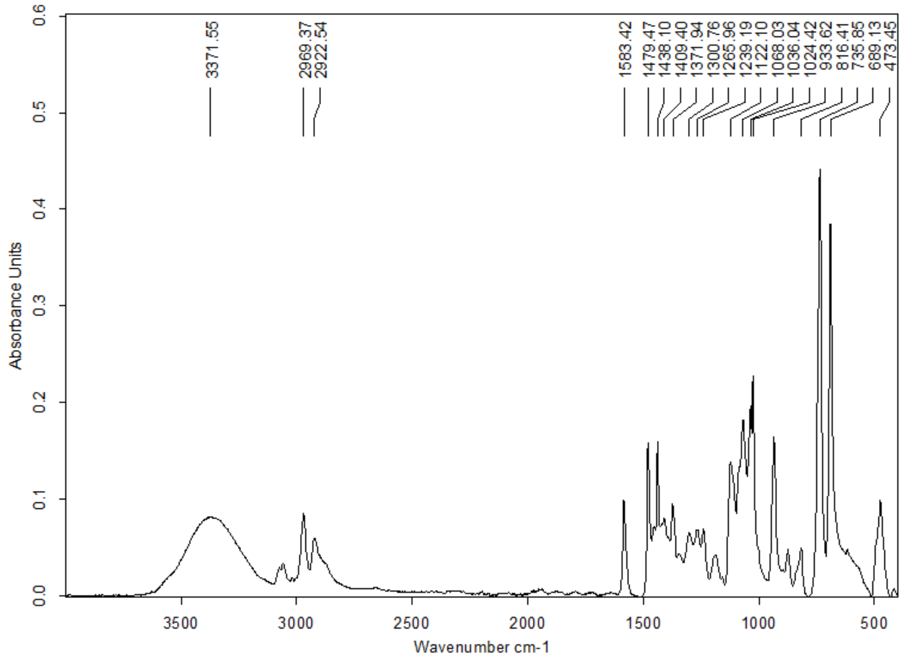

Figure S31. IR spectrum of racemic 1-[(4-methylphenyl)sulfanyl]propan-2-ol (±)-**1b**.

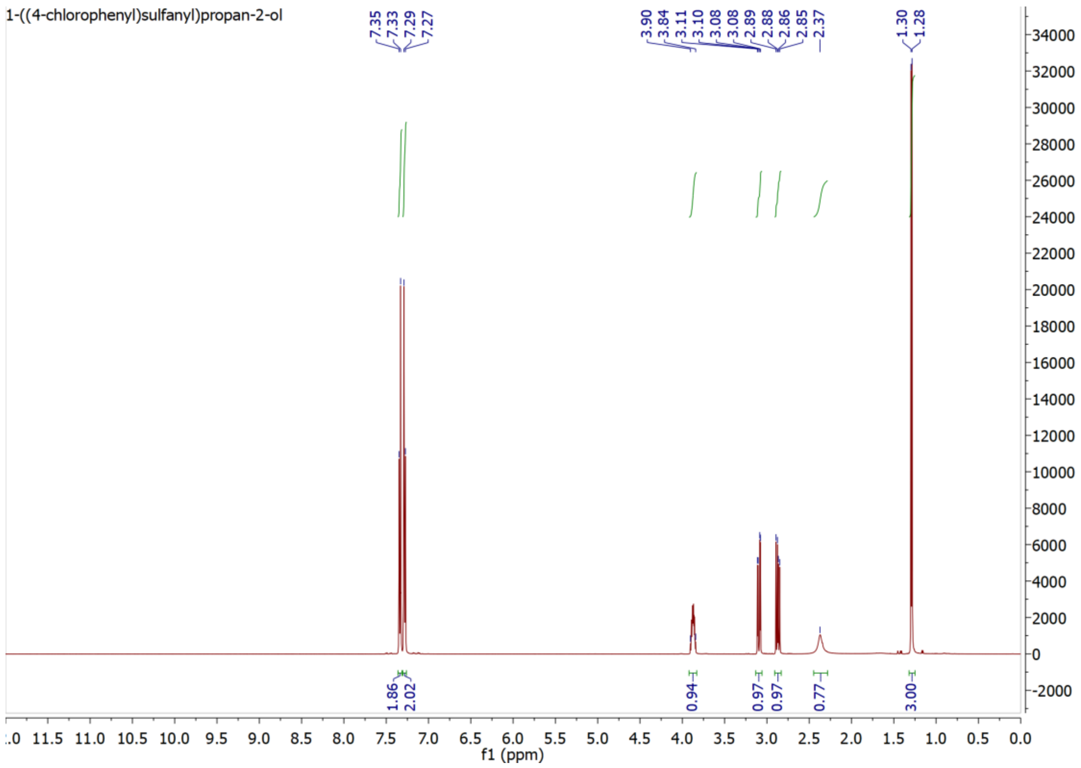

Figure S32. ^{1}H NMR spectrum of racemic 1-[(4-chlorophenyl)sulfanyl]propan-2-ol (±)-**1c**.

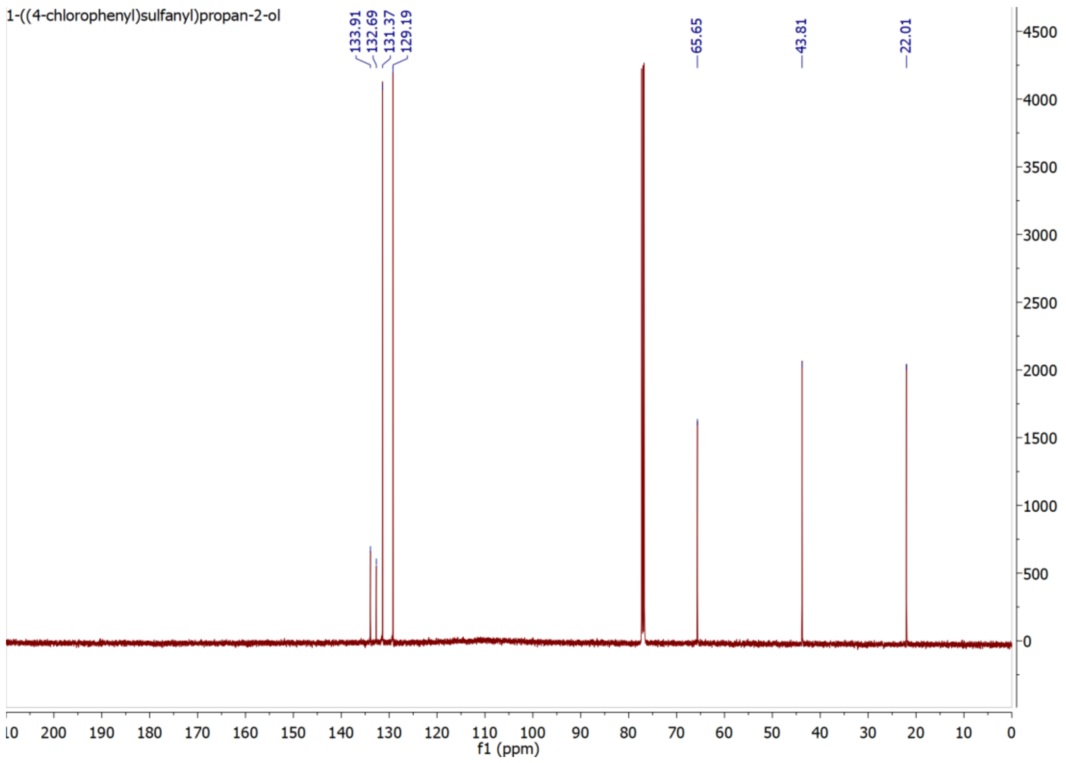

Figure S33. ^{13}C NMR spectrum of racemic 1-[(4-chlorophenyl)sulfanyl]propan-2-ol (±)-**1c**.

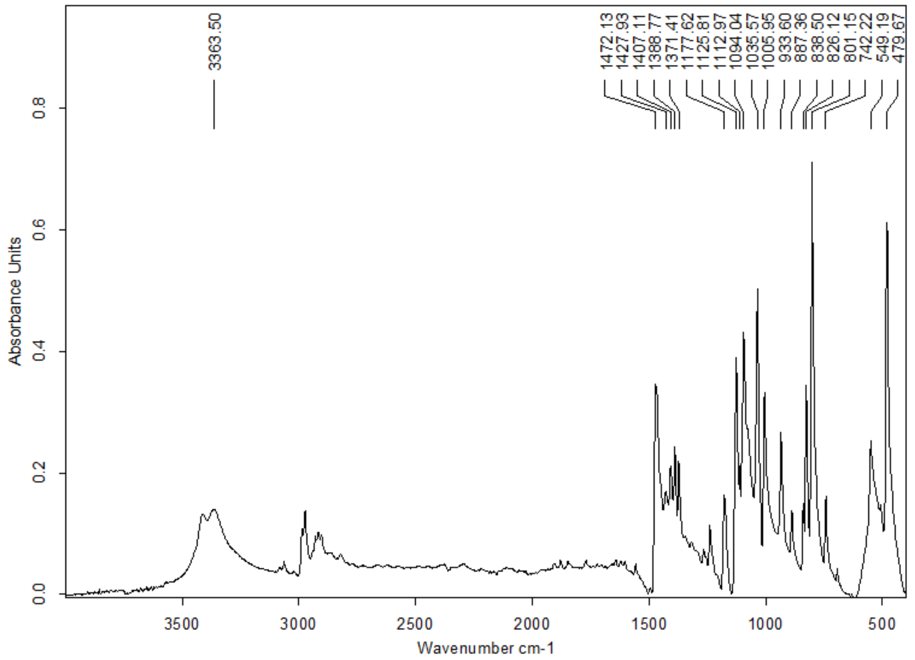

Figure S34. IR spectrum of racemic 1-[(4-chlorophenyl)sulfanyl]propan-2-ol (±)-**1c**

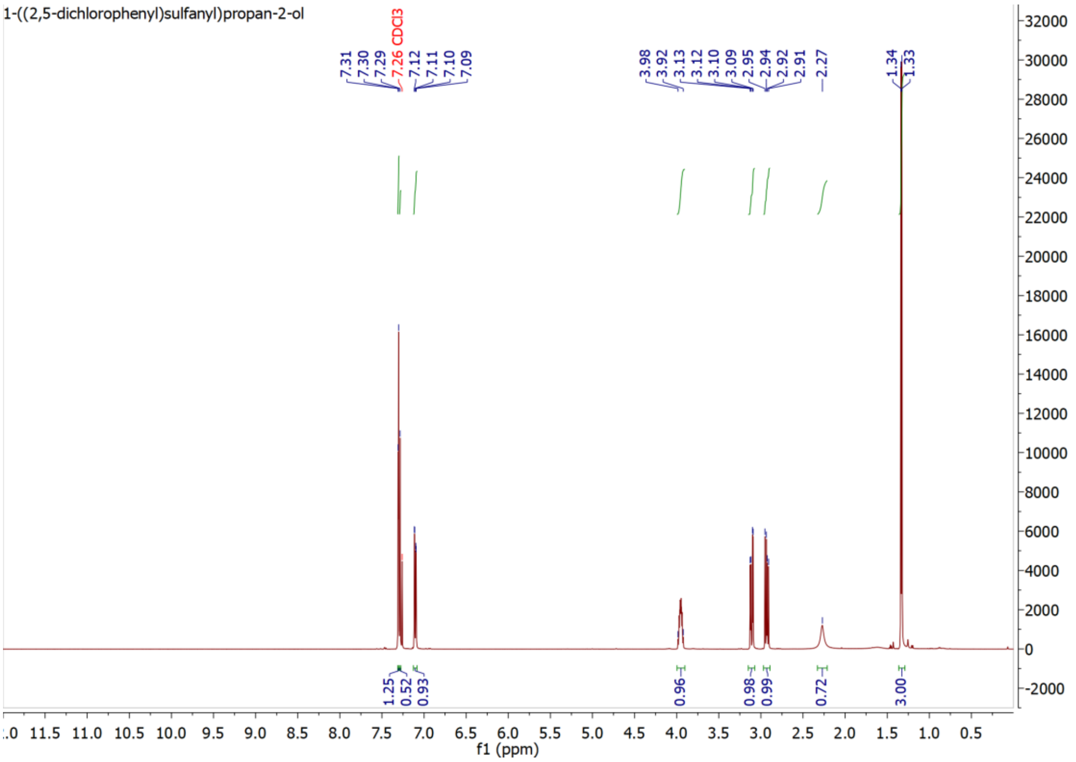

Figure S35. ^{1}H NMR spectrum of racemic 1-[(2,5-dichlorophenyl)sulfanyl]propan-2-ol (±)-**1d**.

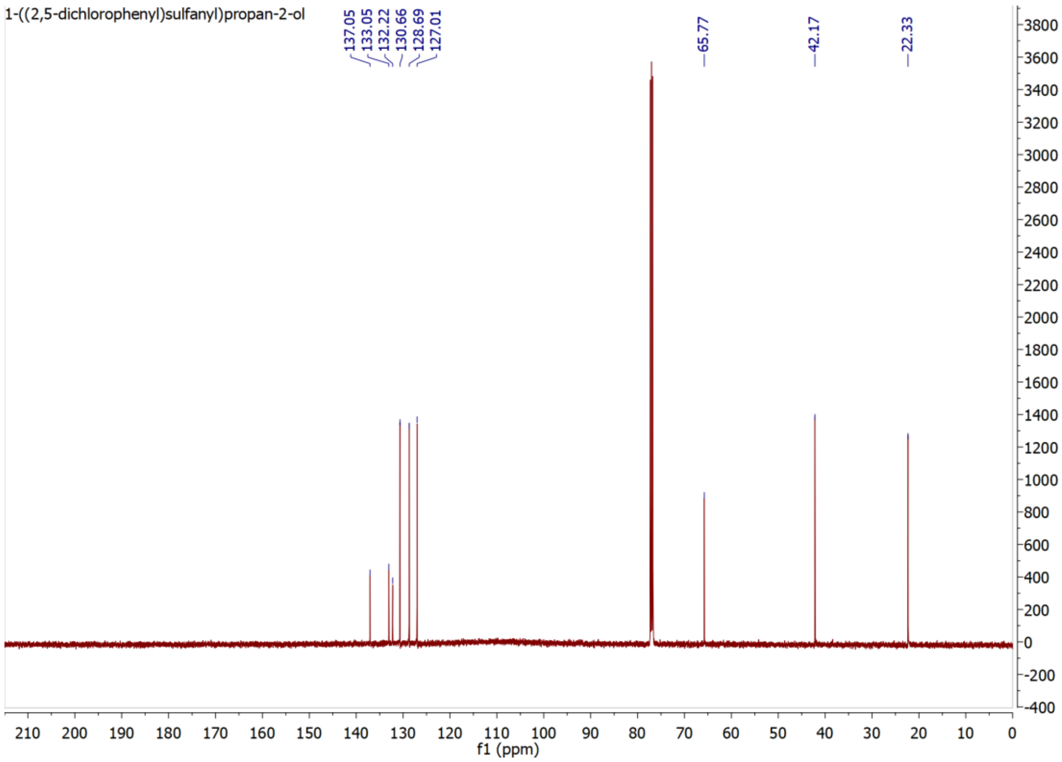

Figure S36. ^{13}C NMR spectrum of racemic 1-[(2,5-dichlorophenyl)sulfanyl]propan-2-ol (±)-**1d**

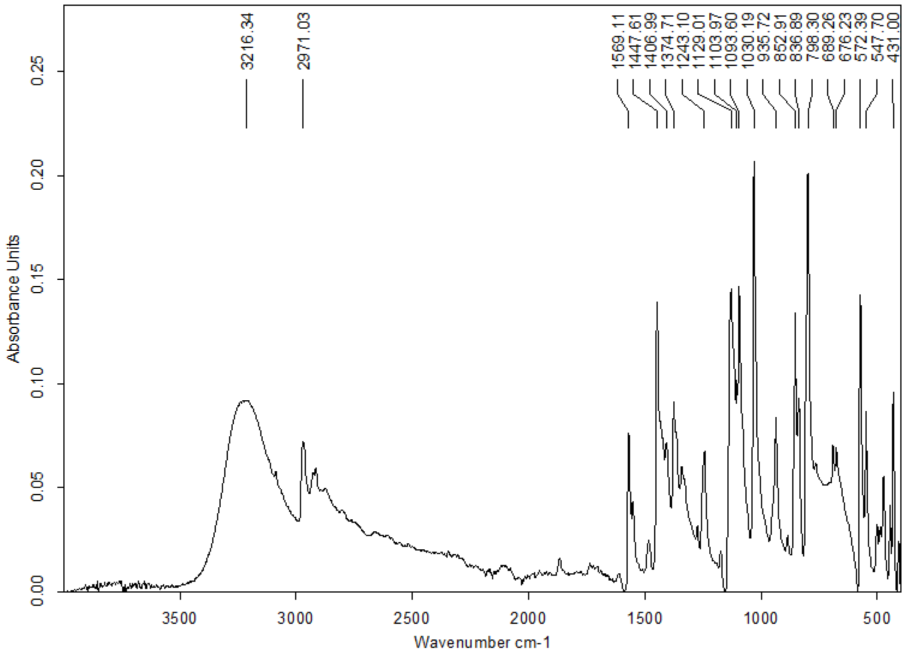

Figure S37. IR spectrum of racemic 1-[(2,5-dichlorophenyl)sulfanyl]propan-2-ol (±)-**1d**.

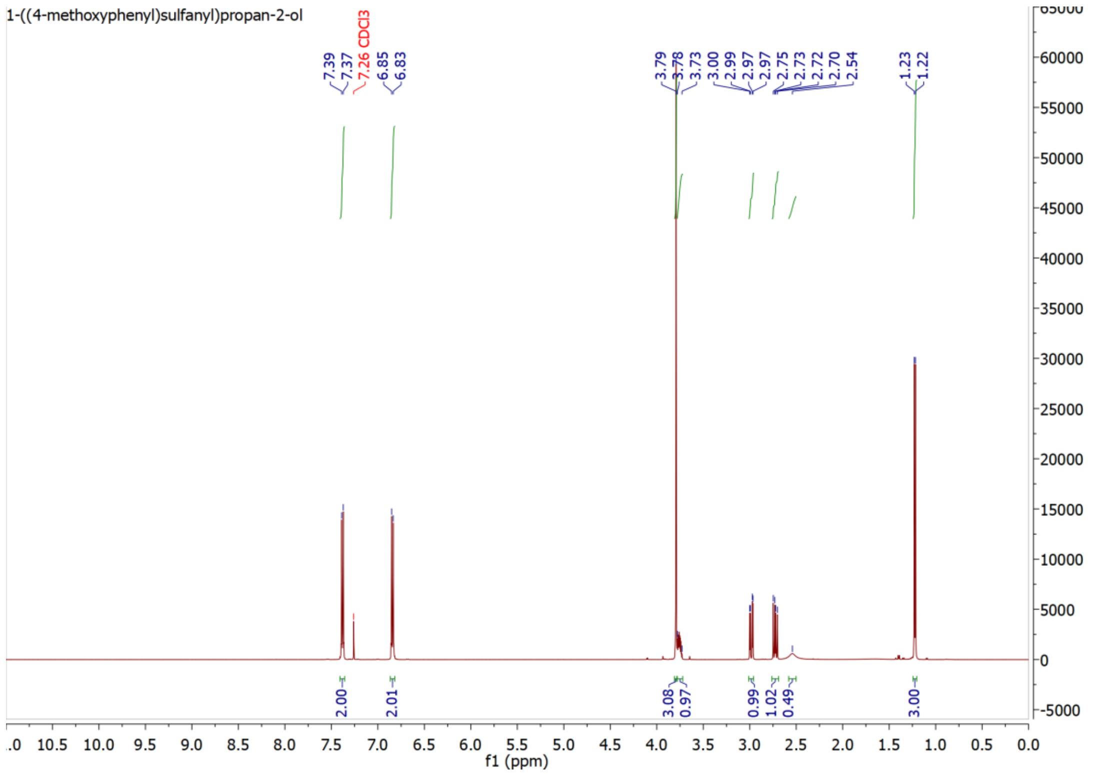

Figure S38. ¹H NMR spectrum of racemic 1-((4-methoxyphenyl)sulfanyl]propan-2-ol (±)-**1e**.

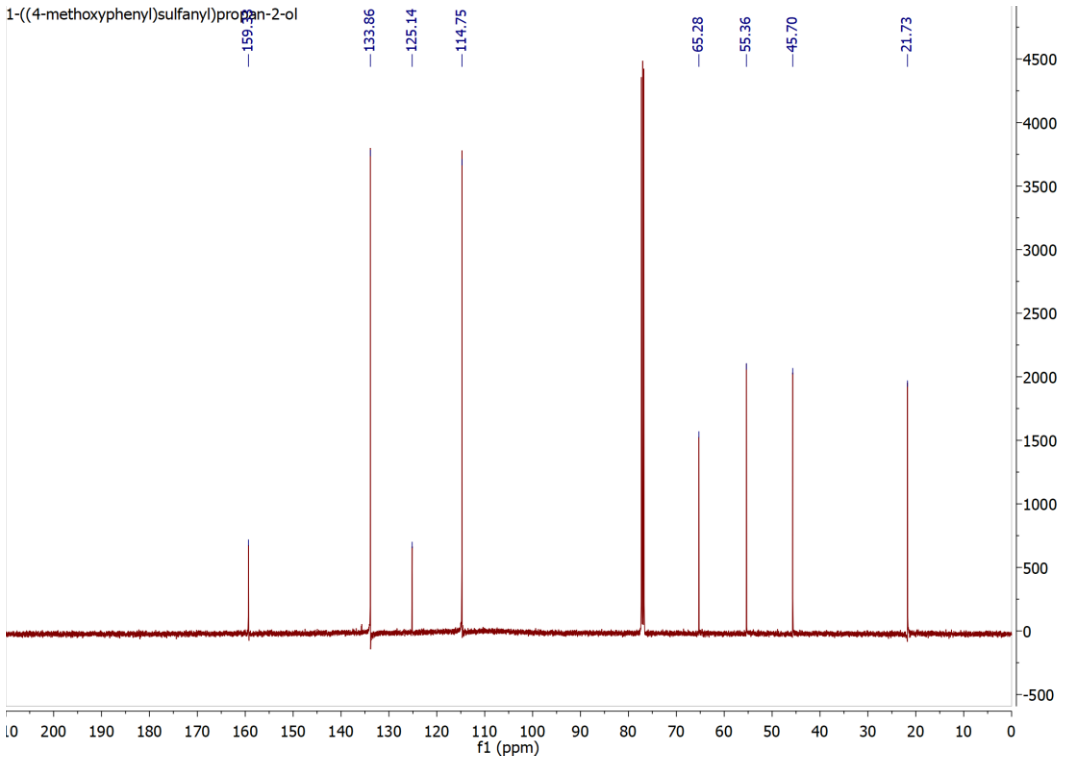

Figure S39. ¹³C NMR spectrum of racemic 1-((4-methoxyphenyl)sulfanyl]propan-2-ol (±)-**1e**.

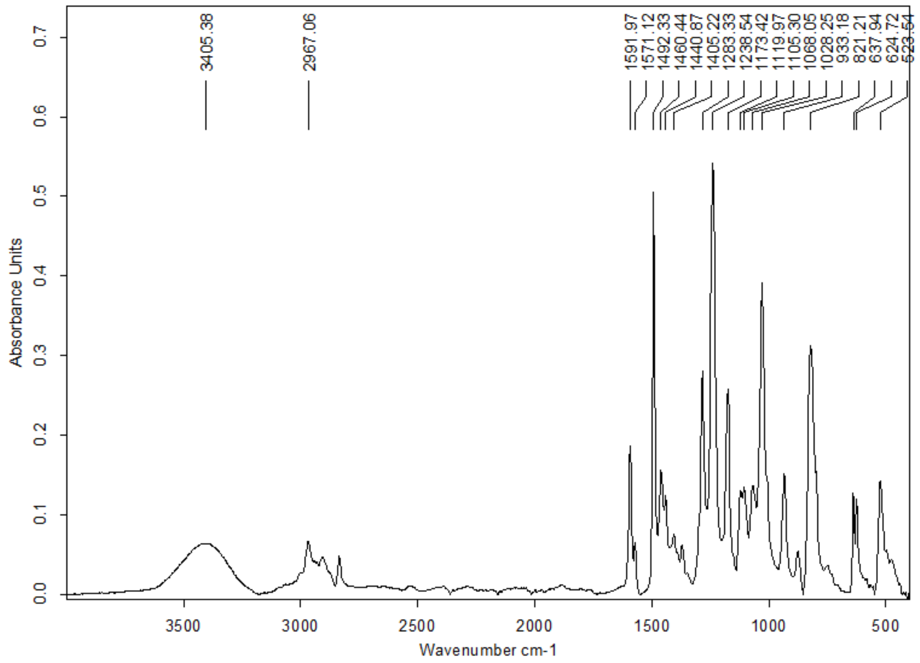

Figure S40. IR spectrum of racemic 1-((4-methoxyphenyl)sulfanyl]propan-2-ol (±)-**1e**.
